# Inhaled siRNA nanoparticles targeting *IL11* inhibit lung fibrosis and improve pulmonary function post-bleomycin challenge

**DOI:** 10.1126/sciadv.abn7162

**Published:** 2022-06-22

**Authors:** Xin Bai, Guolin Zhao, Qijing Chen, Zhongyu Li, Mingzhu Gao, William Ho, Xiaoyang Xu, Xue-Qing Zhang

**Affiliations:** 1 Engineering Research Center of Cell and Therapeutic Antibody, Ministry of Education, and School of Pharmacy, Shanghai Jiao Tong University, 800 Dongchuan Road, Shanghai 200240, PR China.; 2 Department of Chemical and Materials Engineering, New Jersey Institute of Technology, Newark, NJ 07102, USA.; 3 Department of Biomedical Engineering, New Jersey Institute of Technology, Newark, NJ 07102, USA.

## Abstract

Interleukin-11 (IL-11) is a profibrotic cytokine essential for the differentiation of fibroblasts into collagen-secreting, actin alpha 2, smooth muscle–positive (ACTA2^+^) myofibroblasts, driving processes underlying the pathogenesis of idiopathic pulmonary fibrosis (IPF). Here, we developed an inhalable and mucus-penetrative nanoparticle (NP) system incorporating siRNA against *IL11* (si*IL11*@PPGC NPs) and investigated therapeutic potential for the treatment of IPF. NPs are formulated through self-assembly of a biodegradable PLGA-PEG diblock copolymer and a self-created cationic lipid-like molecule G0-C14 to enable efficient transmucosal delivery of si*IL11*. Noninvasive aerosol inhalation hindered fibroblast differentiation and reduced ECM deposition via inhibition of ERK and SMAD2. Furthermore, si*IL11*@PPGC NPs significantly diminished fibrosis development and improved pulmonary function in a mouse model of bleomycin-induced pulmonary fibrosis without inducing systemic toxicity. This work presents a versatile NP platform for the locally inhaled delivery of siRNA therapeutics and exhibits promising clinical potential in the treatment of numerous respiratory diseases, including IPF.

## Teaser

An inhaled siRNA NP exerts antifibrotic effects and improves lung function in pulmonary fibrosis by blocking ERK signaling.

## INTRODUCTION

Idiopathic pulmonary fibrosis (IPF) is a chronic and progressive interstitial lung disease, ultimately leading to a permanent worsening of pulmonary function and death (*1*–*3*). Approximately 5 million people worldwide suffer from IPF with an average median survival of 3 to 5 years (*4*). Environmental insults, side effects of medication, and genetic factors are known to cause IPF (*5*, *6*). Clinical reports have also shown that coronavirus disease 2019 (COVID-19) patients may produce fibrotic responses as a complication after severe acute respiratory syndrome coronavirus-2 (SARS-CoV-2) infection (*7*, *8*). Pirfenidone and nintedanib are the only approved treatments for IPF, yet both of these antifibrotic drugs have notable adverse effects and are merely palliative (*9*, *10*). There is thus an urgent need to identify treatable targets and develop new therapeutic approaches for slowing down or even reversing the disease progression.

Although the mechanisms underlying the pathogenesis of IPF remain poorly understood, many studies have suggested that it is associated with a persistent injury to the alveolar epithelium, which drives dysregulated repair processes and leads to the recruitment of downstream effector cells and secretion of multiple cytokines and fibrotic factors (*11*). The released profibrogenic factors initiate epithelial-to-mesenchymal transition (EMT) and fibroblast-to-myofibroblast transdifferentiation (FMT), contributing to the IPF pathology (*12*). Particularly, myofibroblasts, which are typically activated fibroblasts and represent the predominant mediator of pulmonary fibrosis, deposit excessive extracellular matrix (ECM) components and show resistance to cell apoptosis, causing increased tissue stiffness and aberrant scar formation (*13*–*15*). Interleukins (ILs) have been reported to play diverse roles in fibroblast-related lung fibrosis. For example, IL-1β, IL-13, and IL-5 promote fibrosis, while IL-7, IL-37, and, IL-27 could conversely alleviate fibrosis (*12*, *16*–*22*). Recent evidence identified IL-11 as a potent profibrotic cytokine involved in various types of fibrotic diseases (*23*, *24*). The binding of IL-11 and its heterodimeric receptor subunits, IL-11RA and glycoprotein 130 (gp130), triggers fibroblast activation and ECM production via the extracellular signal–regulated kinase (ERK) signaling pathway (*25*). Therefore, the down-regulation of IL-11 might be a potential therapeutic approach for the treatment of pulmonary fibrosis.

Since the first small interfering RNA (siRNA)–based drug (patisiran) was approved to treat hereditary transthyretin-mediated (hATTR) amyloidosis by the U.S. Food and Drug Administration (FDA), nanocarrier-assisted siRNA therapeutics have achieved a critical breakthrough in the clinic and generated widespread interest (*26*, *27*). Lipid- and polymer-based nanocarriers have demonstrated the outstanding merits of siRNA delivery including tunable physiochemical properties, high encapsulation efficiency, protection from enzymatic degradation, improved cell permeability, and diverse surface modifications, making them attractive carriers for effective delivery of siRNA therapeutics to the liver and the other target organs in a safe and reproducible manner (*28*–*31*). Direct delivery of siRNA therapeutics to the lungs through a pulmonary route is promising for the treatment of a range of respiratory diseases such as cystic fibrosis, asthma, and the global pandemic caused by SARS-CoV-2 (*32*). As a frequently used route for drug administration in the clinic, noninvasive inhalation focuses the therapeutics locally in the lungs and allows for drug deposition throughout the whole bronchiolar and alveolar epithelium with improved compliance and reduced systemic exposure, providing new paradigms for the management of respiratory diseases (*32*, *33*). Aerosolized ALN-RSV01 was the first clinically tested pulmonary deliverable siRNA therapeutic targeting the respiratory syncytial virus (RSV) nucleocapsid protein. It was demonstrated to effectively reduce the incidence of new or progressive bronchiolitis obliterans syndrome in lung transplant patients with RSV infection following inhalation (*34*). Despite the progress made, there are no inhaled RNA therapeutics approved for use in clinics to date. This is due to certain hurdles inherent to the nebulization and inhalation of the RNA therapeutics; inhaled siRNA therapy usually requires the development of carriers that can withstand the drastic shearing forces generated during nebulization, be concentrated into small volumes, traverse the mucus, and reach the deep lung (*35*, *36*).

**Fig. 1.**
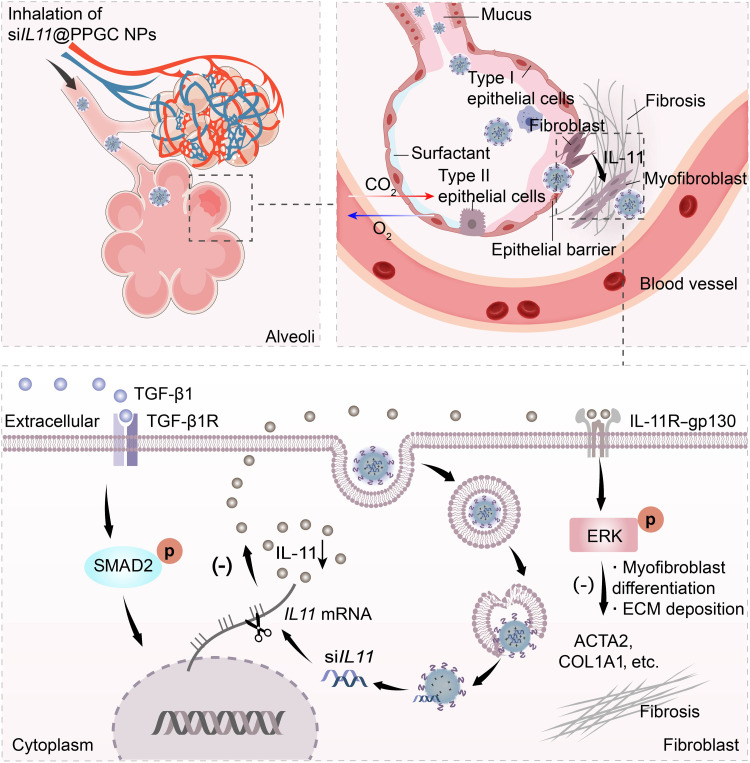
Inhaled delivery of siRNA-encapsulated PPGC NPs to MLFs for the treatment of IPF.

**Fig. 2.**
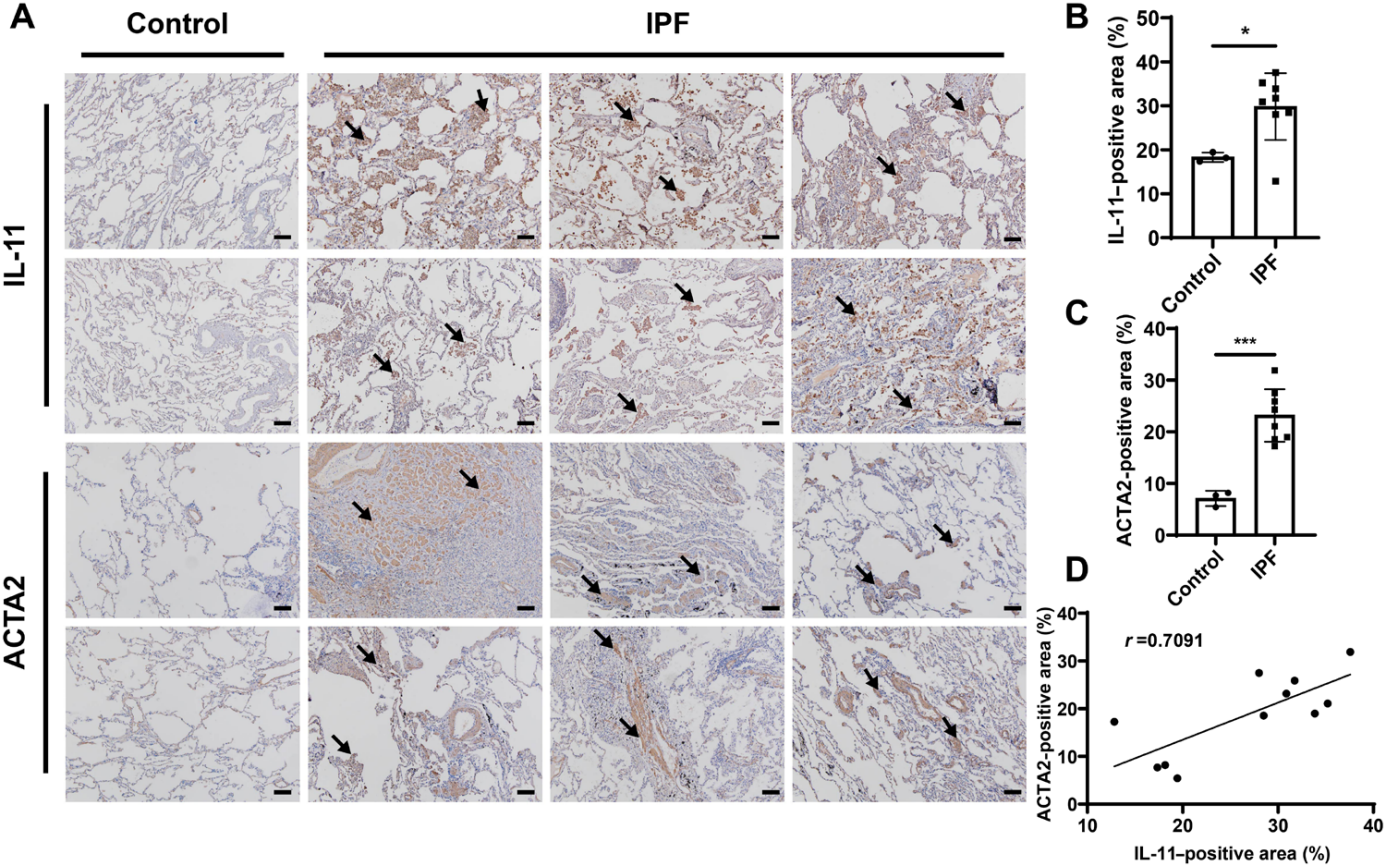
Immunohistochemistry staining of human samples. (**A**) Representative images of IL-11 and ACTA2 immunohistochemistry staining from IPF patients (*N* = 8) and healthy controls (*N* = 3). 3,3′-Diaminobenzidine (DAB)–positive regions are indicated by the arrows. Scale bars, 100 μm. (**B** and **C**) Quantification of IL-11–positive area (B) and ACTA2-positive area (C). (**D**) Correlation analysis between IL-11– and ACTA2-positive area (*N* = 11). Significant differences were assessed using a two-tailed unpaired Student’s *t* test (B and C). Correlation coefficient (*r*) was assessed using a nonparametric Spearman correlation analysis (D). Results are presented as means ± SD. *P < 0.05, ***P < 0.001.

Here, we demonstrate a proof of concept for the design and preclinical use of an inhalable nanoparticulate-mediated RNA interference (RNAi) approach targeting *IL11* for inhibition of lung fibrosis progression and enhanced recovery of pulmonary function in a mouse model of bleomycin-induced pulmonary fibrosis (Fig. 1). First, a significant up-regulation of IL-11 and actin alpha 2, smooth muscle (ACTA2) in lung tissues from both IPF patients and the mouse model of bleomycin-induced pulmonary fibrosis was observed, and a strong correlation was identified by quantitative analysis of the immunohistochemistry images (Fig. 2). Our finding is consistent with recent research that revealed the prominent role of IL-11 signaling in pulmonary fibrosis pathology (*23*), suggesting that IL-11 could serve as a potential drug target for the treatment of lung fibrosis and IPF. We then constructed nanoparticles (NPs) from (i) our previously reported cationic lipid-like molecule designated “G0-C14” to facilitate entrapment of siRNA therapeutics and (ii) a biodegradable poly(lactide-*co*-glycolide)-*b*-poly(ethylene glycol) (PLGA-PEG) diblock copolymer to form solid NPs that protect siRNA from nuclease degradation and facilitate transmucosal delivery of siRNA against *IL11* (si*IL11*). The NPs loaded with si*IL11*, termed as si*IL11*@PPGC NPs, were then nebulized to treat an experimental murine fibrosis model using a vibrating mesh nebulizer. The locally released si*IL11* potently hindered fibroblast differentiation and diminished ECM deposition while blocking SMAD family member 2 (SMAD2) and ERK activation. Inhaled si*IL11*@PPGC NPs significantly promoted pulmonary function recovery in the murine fibrosis model as evidenced by the increased compliance (Crs), forced vital capacity (FVC), and forced expiratory volume at 0.2 s (FEV0.2) and decreased resistance (Rrs) and elastance (Ers). In this work, we first demonstrated the use of inhalable NPs incorporating siRNA to block IL-11 signaling in a preclinical model of pulmonary fibrosis and showed great therapeutic potential of this strategy for repairing injured lung tissues.

## RESULTS

### Immunohistochemistry staining

ACTA2, a hallmark of myofibroblasts, is an actin isoform facilitating cell contraction and migration and plays an important role in fibrogenesis (*37*, *38*). We first incubated mouse lung fibroblasts (MLFs) with various concentrations of transforming growth factor–β1 (TGF-β1) and found that TGF-β1 could induce relatively high *IL11* expression even at concentrations as low as 10 ng/ml (fig. S1). Therefore, TGF-β1 with a concentration of 10 ng/ml was used to perform the following downstream cell experiments. We then examined the expression of IL-11 and ACTA2 in lung sections from IPF patients and the mouse model of bleomycin-induced pulmonary fibrosis. The immunohistochemistry staining showed that IL-11 and ACTA2 were hardly detected in the samples from the control group but markedly expressed in the samples from both IPF patients (Fig. 2, A to C) and experimental murine models (fig. S2). A linear regression model was established on the basis of the quantitative analysis of IL-11– and ACTA2-positive area in immunohistochemistry staining images, which indicated that the expression of the fibrosis marker ACTA2 was positively correlated with IL-11 level, with a calculated correlation coefficient of 0.7091 (Fig. 2D). This finding suggests a preeminent role for IL-11 in lung fibrosis formation and implies that inhibition of IL-11 could be a promising strategy for hindering fibrosis development.

### Preparation and characterization of PPGC NPs

**Fig. 3.**
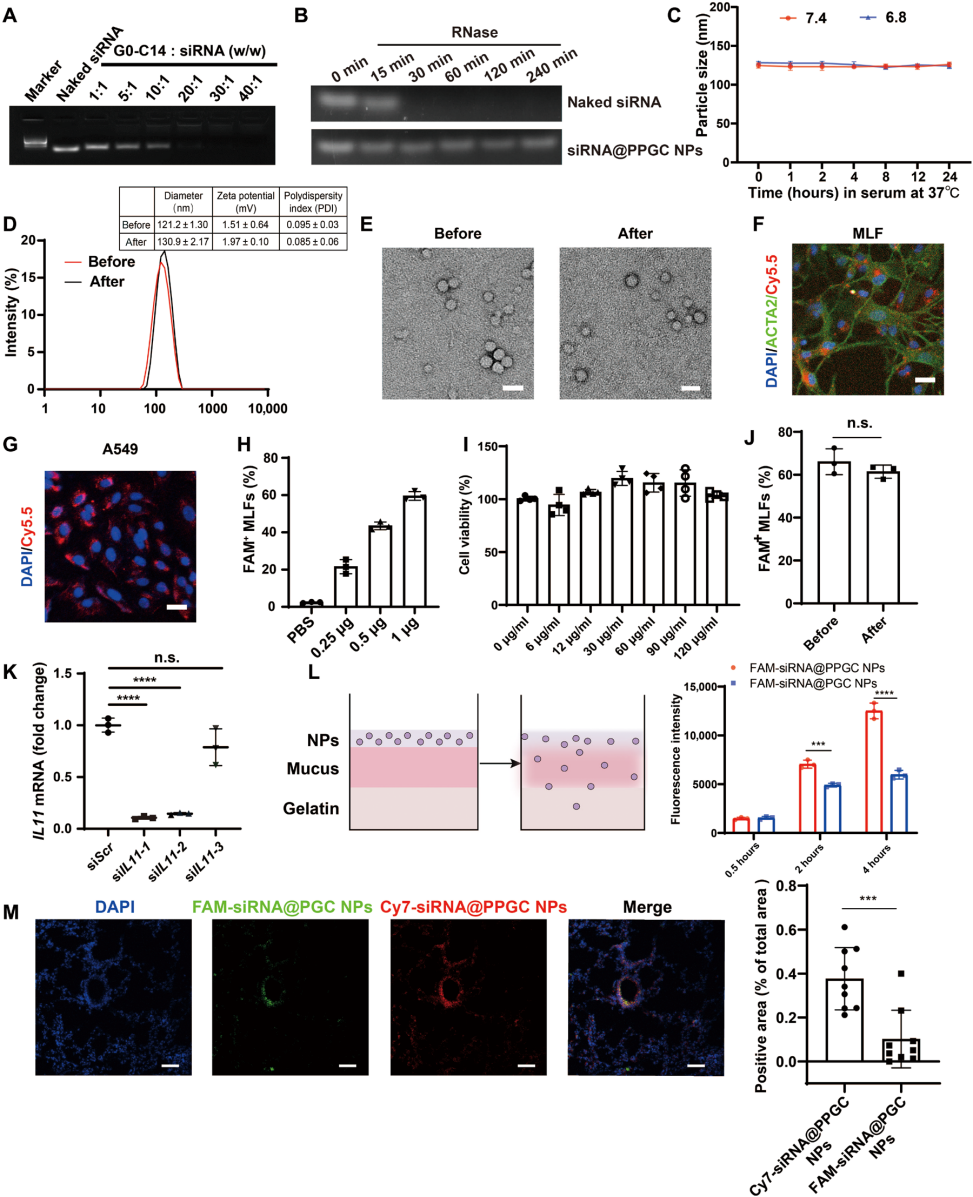
Preparation and characterization of PPGC NPs. (**A**) Investigation of interaction between G0-C14 and siRNA. (**B**) Stability of naked siRNA and NP-encapsulating siRNA against RNase. (**C**) Size of PPGC NPs measured in PBS containing 10% FBS at pH 7.4 or 6.8 at 37°C for 24 hours (*N* = 3). (**D**) Size measurements of PPGC NPs before and after nebulization. (**E**) Representative TEM image of PPGC NPs. Scale bars, 50 nm. (**F** and **G**) Cellular uptake of Cy5.5-labeled NPs in MLFs (F) and A549 (G). Red, blue, and green fluorescence indicates NPs, nucleus, and ACTA2, respectively. Scale bars, 25 μm. (**H**) Dose-dependent cellular uptake of FAM-siRNA@PPGC NPs in MLFs (*N* = 3). (**I**) Cell viability of MLFs treated with NPs at different concentrations (*N* = 4). (**J**) Impact of the nebulization process on cellular uptake of FAM-siRNA@PPGC NPs in the primary MLFs (*N* = 3). (**K**) Silencing efficiency comparison of si*IL11*@PPGC NPs in MLFs (*N* = 3). (**L**) Penetration of NPs with (FAM-siRNA@PPGC NPs) and without (FAM-siRNA@PGC NPs) PEG coating in an artificial mucus model (*N* = 3). (**M**) In vivo penetration of NPs in airway mucus (*N* = 3). For each mouse, three different fields of the sections acquired from the same position of the lungs were imaged and analyzed for a total of nine measurements. Scale bars, 50 μm. Significant differences were assessed using a two-tailed unpaired Student’s *t* test (J and M), a one-way analysis of variance (ANOVA) with Tukey test (K), and a two-way ANOVA with Tukey test (L). Results are presented as means ± SD. ***P < 0.001, ****P < 0.0001, n.s., not significant, *P* > 0.05.

PLGA-PEG was synthesized according to our previous descriptions (*39*). G0-C14 was synthesized by reacting 1,2-epoxytetradecane with generation 0 of ethylenediamine core–poly(amidoamine) (PAMAM) dendrimer as described previously (*39*), but with significant modification and optimization in ratios of reagents used in the G0-C14 synthesis. The chemical structures of PLGA-PEG and G0-C14 were characterized by ^1^H nuclear magnetic resonance (NMR) (CDCl_3_, 400 MHz) as shown in figs. S3 and S4. The PPGC NPs were prepared using G0-C14 that enabled nucleic acid complexation and PLGA-PEG that was leveraged for making a stable NP core via a robust self-assembly method. Figure 3A showed that siRNA could be effectively condensed by G0-C14 at a weight ratio of 1:20 or above, with no leaching of siRNA observed from electrophoresis. An siRNA/G0-C14 weight ratio of 1:30 was chosen for NP preparation in the following experiments.

To verify the protective effect of PPGC NPs on siRNA, naked siRNA or siRNA encapsulated within PPGC NPs (siRNA@PPGC NPs) was incubated with ribonuclease (RNase) for different time durations (0, 15, 30, 60, 120, and 240 min). The naked siRNA degraded rapidly, while the siRNA extracted from PPGC NPs retained structural integrity when exposed to RNase for up to 4 hours (Fig. 3B). We also investigated the impact of pH on the stability of PPGC NPs, and the dynamic light scattering (DLS) result showed that there were no significant changes in particle size when incubating NPs in phosphate-buffered saline (PBS) containing 10% fetal bovine serum (FBS) at pH 7.4 or 6.8 within 24 hours, suggesting that the PPGC NPs could maintain intact structure in the slightly acidic extracellular pH of IPF lung tissues (Fig. 3C) (*40*). To mimic physiological extracellular and acidic endosomal environments, siRNA release profiles from PPGC NPs were determined in PBS at pH 7.4 and 5.0. The PPGC NPs exhibited faster release of siRNA at pH 5.0 than at pH 7.4 (~81.1% at pH 5.0 and ~60.0% at pH 7.4 after 120 hours) (fig. S5). We then examined whether the drastic shearing forces generated during the nebulization procedure could damage the NP structure. The DLS results showed that no remarkable change was observed in terms of the average size of siRNA@PPGC NPs before and after nebulization (Fig. 3D), and the morphology of siRNA@PPGC NPs remained spherical as characterized by transmission electron microscopy (TEM) (Fig. 3E). The fluorescence assay implies that there is no significant difference when comparing siRNA encapsulation efficiencies within NPs before and after nebulization (93.31 versus 93.12%) (fig. S6). The tolerance of PPGC NPs against the drastic shearing forces during the nebulization process makes them an ideal candidate for aerosol delivery.

The cellular uptake of the PPGC NPs was assessed in both primary MLFs and A549, and pronounced uptake was observed in confocal images (Fig. 3, F and G). When fluorescein amidite (FAM)–labeled siRNA (FAM-siRNA) was encapsulated within PPGC NPs (FAM-siRNA@PPGC NPs) followed by incubation with MLFs at various concentrations, a dose-dependent cellular internalization of FAM-siRNA was observed from the flow cytometry analysis, and no obvious in vitro cytotoxicity was observed in the range of tested concentrations, even at the highest NP concentration of 120 μg/ml (Fig. 3, H and I). We also investigated the impact of nebulization process on NP uptake in the primary MLFs. The results showed that there is no significant difference in cellular uptake of NPs before and after nebulization (Fig. 3J). To determine the cellular uptake mechanism of siRNA@PPGC NPs, we tested the cell internalization of FAM-siRNA@PPGC NPs in MLFs pretreated with different inhibitors using flow cytometry. Figure S7 demonstrated a drastic decrease of uptake from ~60% (without inhibitor) to ~30% in the presence of chlorpromazine (CPZ), while no changes were observed for filipin and EIPA 5-(N-Ethyl-N-isopropyl)-Amiloride, suggesting that cellular internalization of siRNA@PPGC NPs is mainly mediated by clathrin.

We then encapsulated three predesigned si*IL11*s, termed si*IL11*-1, si*IL11*-2, and si*IL11*-3, within these biocompatible PPGC NPs (si*IL11*@PPGC NPs), respectively, and tested the silencing efficiency of these NPs by measuring *IL11* mRNA expression in MLFs. As shown in Fig. 3K, quantitative reverse transcription polymerase chain reaction (qRT-PCR) result demonstrated that treatment of the NPs incorporated with si*IL11-1* resulted in the lowest *IL11* mRNA expression, which was reduced by nearly 90% compared with the control group. Hence, si*IL11-1*–loaded PPGC NPs were used in the subsequent experiments.

As shown in Fig. 3L, an artificial mucus model was used to investigate the impact of PEG coating on the transmucosal penetration capability of fluorescently labeled NPs (*41*). Briefly, FAM-siRNA was encapsulated into PPGC NPs to yield FAM-siRNA@PPGC NPs, which has PEG coating. We then encapsulated FAM-siRNA into NPs consisting of PLGA and G0-C14 to yield FAM-siRNA@PGC NPs without the PEG coating while keeping the other components, to serve as a control. The surface zeta potential of both PGC NPs and PPGC NPs decreased from approximately 15 mV to nearly neutral after siRNA encapsulation (fig. S8). The resulting NPs (with or without PEG coating) at equivalent dose of FAM-siRNA were added to the artificial mucus, and the fluorescence intensity of the agarose gel layer after penetration was analyzed. As shown in Fig. 3L, higher intensity of FAM signal was detected in the group treated with FAM-siRNA@PPGC NPs than with FAM-siRNA@PGC NPs, suggesting that the transmucosal penetration ability of NPs was significantly enhanced by PEG coating. In addition, the magnitude of enhancement in NP penetration markedly increased with incubation time.

To compare the transmucosal penetration of NPs with and without PEG coating in vivo, FAM-siRNA@PGC NPs and Cy7-siRNA@PPGC NPs were simultaneously administered in healthy mice via intratracheal injection, and lung tissues were collected and stained for the visualization of NP localization. Figure 3M illustrated that more Cy7-siRNA@PPGC NPs accumulated in bronchiole and alveoli than FAM-siRNA@PGC NPs. The in vivo distribution of Cy7-siRNA@PPGC NPs in mouse lungs was depicted in fig. S9. The results suggested that PPGC NPs with PEG coating exhibited excellent penetration and retention ability, making it a suitable vector for inhalable siRNA therapy.

### si*IL11*@PPGC NPs inhibit MLF differentiation and migration

**Fig. 4.**
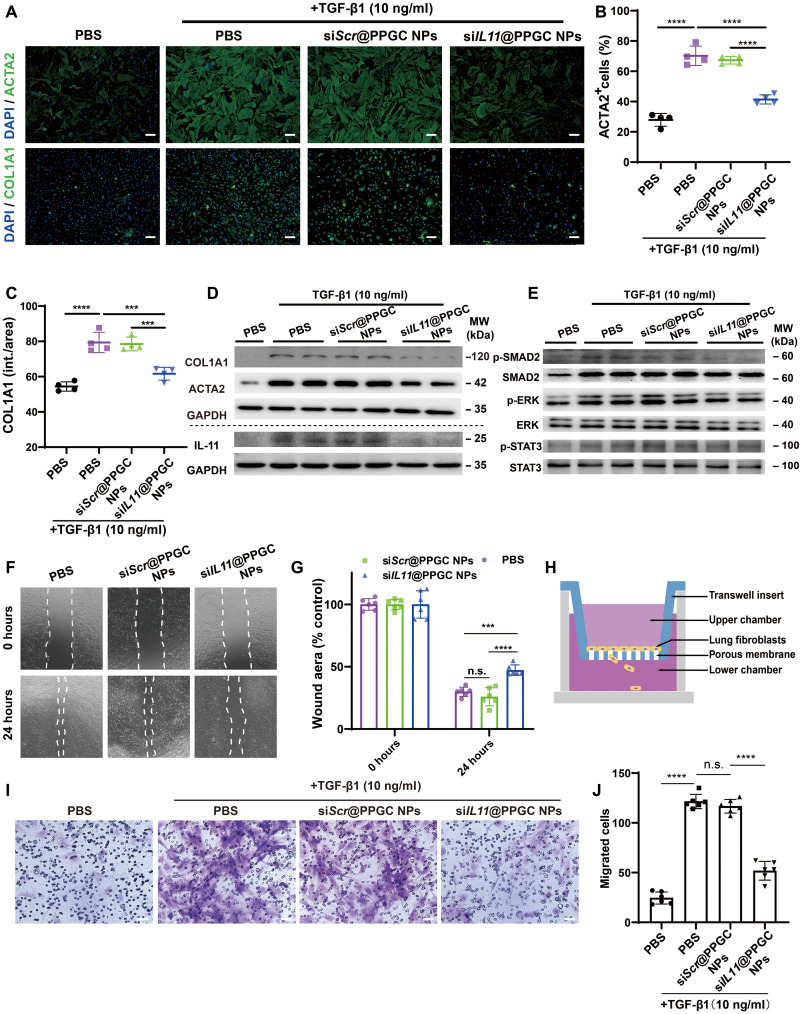
si*IL11*@PPGC NPs inhibit fibroblast activation and migration. (**A**) Representative images of ACTA2 and COL1A1 immunostaining in MLFs treated with TGF-β1 (10 ng/ml) for 24 hours in the presence of PBS, si*Scr*@PPGC NPs, or si*IL11*@PPGC NPs. MLFs treated with PBS only was used as a negative control. Scale bars, 100 μm. (**B** and **C**) Quantification analysis of ACTA2^+^ cells (B) and COL1A1 immunostaining (intensity/area) (C) in MLFs treated with TGF-β1 for 24 hours in the presence of si*Scr*@PPGC NPs or si*IL11*@PPGC NPs (*N* = 4). (**D** and **E**) Western blotting of COL1A1, ACTA2, IL-11, phosphorylation, and total expression of SMAD2, ERK, and STAT3 in MLFs in the presence of PBS, si*Scr*@PPGC NPs, or si*IL11*@PPGC NPs followed by TGF-β1 treatment (10 ng/ml) for 24 hours (*N* = 2). GAPDH was used as a housekeeping standard. MW, molecular weight. (**F** and **G**) Representative images of wound-healing assay in MLFs treated with PBS, si*Scr*@PPGC NPs, or si*IL11*@PPGC NPs (*N* = 3). Two fixed fields per well were imaged, and three independent wells were measured with a total of six measurements. (**H**) Schematic illustration of transwell migration assay. (**I** and **J**) Images and quantification of stained cells migrating from the upper chamber to the lower chamber after treatment of PBS, PBS + TGF-β1, si*Scr*@PPGC NPs + TGF-β1, or si*IL11*@PPGC NPs + TGF-β1 (*N* = 3). Images from two different fields per well were acquired, and three independent wells were measured with a total of six measurements. Significant differences were assessed using a one-way ANOVA with Tukey test (B, C, and J) and a two-way ANOVA with Tukey test (G). Results are presented as means ± SD. ***P < 0.001, ****P < 0.0001, n.s., not significant, *P* > 0.05.

We next investigated whether si*IL11*@PPGC NPs could inhibit TGF-β1–induced fibroblast differentiation and ECM production in MLFs. MLFs were treated with scrambled siRNA (si*Scr*)–loaded PPGC NPs (si*Scr*@PPGC NPs) or si*IL11*@PPGC NPs for 4 hours followed by immunofluorescence staining analysis for ACTA2 and collagen type I alpha 1 chain (COL1A1), which are biomarkers of myofibroblasts and the main component of ECM, respectively. Laser scanning confocal microscopy (LSCM)–based analysis demonstrated that si*IL11*@PPGC NP treatment markedly reduced TGF-β1–induced ACTA2 and COL1A1 expression (Fig. 4, A to C).

Consistent with the immunofluorescence results, Western blotting also exhibited a decrease in the expression of IL-11, ACTA2, and COL1A1 at the protein level after si*IL11*@PPGC NP treatment compared with PBS (Fig. 4D). To probe the possible signaling pathways responsible for the fibroblast activation and ECM production, Western blotting was performed to investigate canonical signal transducer and activator of transcription 3 (STAT3), noncanonical ERK, and SMAD2 signaling pathways. This result implied that fibroblast activation and ECM production were triggered in an ERK- and SMAD2-dependent manner. si*IL11*@PPGC NP treatment notably inhibited the IL-11–dependent SMAD2 and ERK phosphorylation, whereas phosphorylated STAT3 (p-STAT3) remained elevated (Fig. 4E). A semiquantitative analysis of Fig. 4 (D and E) has been provided in fig. S10.

Considering that fibrosis development also involves the migration of fibroblasts and myofibroblasts into fibroblastic foci (*42*), we tested the impact of si*IL11*@PPGC NPs on the migration of fibroblasts via both wound-healing assay and transwell migration assay. For wound-healing assay, MLFs were treated with PBS, si*Scr*@PPGC NPs, or si*IL11*@PPGC NPs for 4 hours, and images of the healing process were taken at 24 hours. As shown in Fig. 4 (F and G), wound healing percentage was remarkably decreased in the si*IL11*@PPGC NPs cohort versus the other two cohorts at 24 hours. For transwell migration assay, cells seeded in the Boyden chambers were treated with si*Scr*@PPGC NPs or si*IL11*@PPGC NPs, respectively, followed by incubation with staining solution for the visualization of migrated cells (Fig. 4H). Figure 4 (I and J) showed that a reduction in TGF-β1–driven fibroblast migration was observed in si*IL11*@PPGC NP–treated MLFs. The results revealed the critical role of IL-11 in fibroblast migration and that targeting down-regulation of IL-11 with inhalable siRNA therapeutics could effectively impede fibrosis development by inhibiting cell migration into the fibroblastic foci.

### Transfection efficiency and biodistribution of Cy5.5-labeled NPs after inhalation

**Fig. 5.**
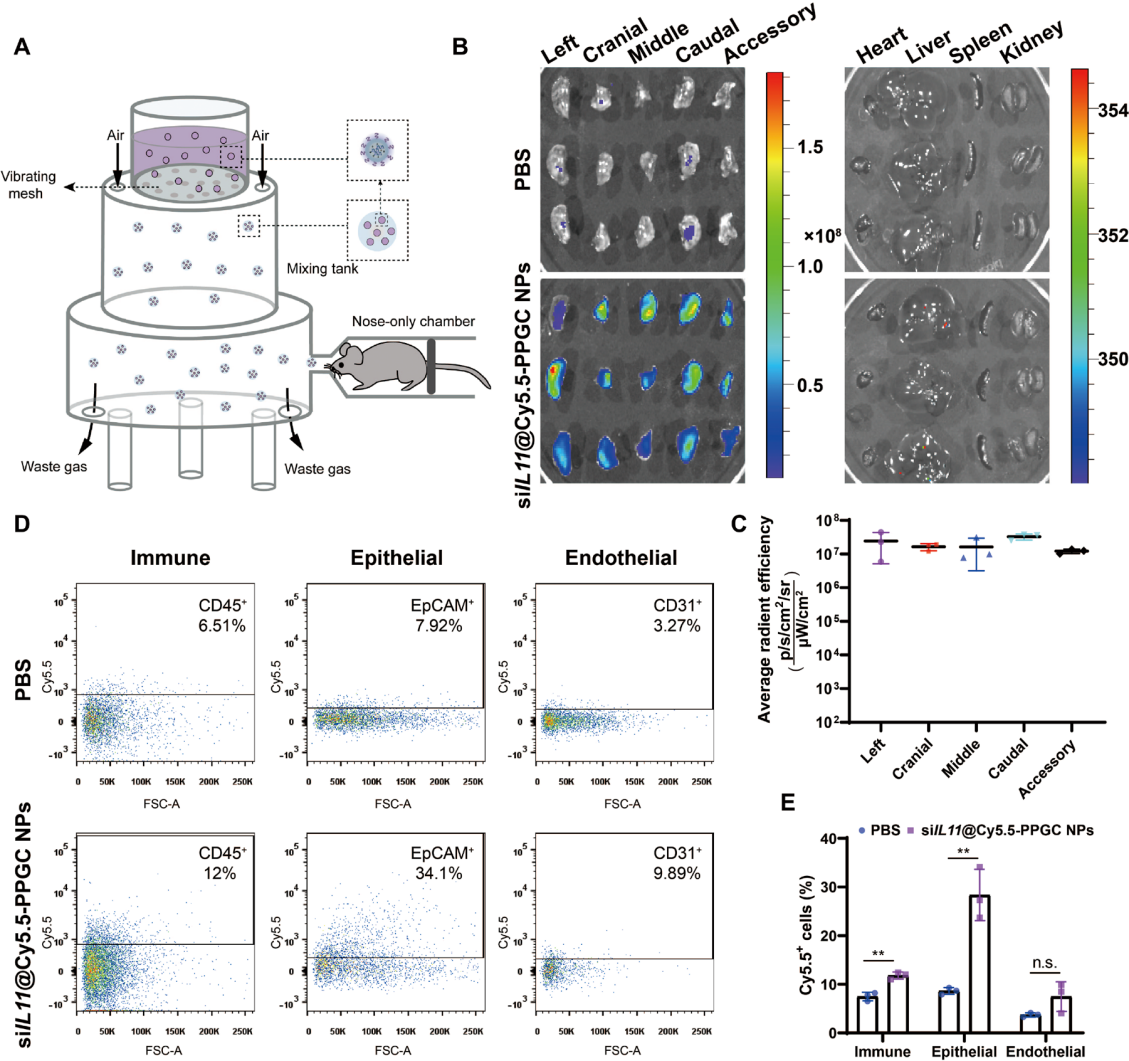
Biodistribution of si*IL11*@PPGC NPs in the lungs of bleomycin-induced fibrosis mice after nebulization. (**A**) Schematic illustration of the vibrating mesh nebulizer device. (**B**) IVIS imaging of lungs and other organs including heart, liver, spleen, and kidney collected at 24 hours after nebulization of Cy5.5-labeled si*IL11*@PPGC NPs. Organs from the mice that received PBS treatment were imaged as control. Fluorescence could be observed in all five lobes (*N* = 3). (**C**) Quantitative analysis of fluorescence signal in left, cranial, middle, caudal, and accessory lobes (*N* = 3). (**D** and **E**) Subcellular localization of si*IL11*@PPGC NPs analyzed by flow cytometry (D) and its quantitative assessment (E). Different cell subtypes were marked with corresponding antibodies including anti-CD31 (endothelial), anti-EpCAM (epithelial), and anti-CD45 (immune) (*N* = 3). Significant differences were assessed using a two-tailed unpaired Student’s *t* test. Results are presented as means ± SD. **P < 0.01, n.s., not significant, *P* > 0.05.

To determine the expression of RNA-based therapeutics throughout the lung following aerosol delivery, Cy5.5-labeled PPGC NPs loaded with m*Luc* (m*Luc*@PPGC NPs) were nebulized to mouse lungs, and the organs were harvested 24 hours after nebulization and immediately imaged with an IVIS system. Figure 5A demonstrates the schematic illustration of the vibrating mesh nebulizer device used in animal studies. As depicted in fig. S11 (A and B), a strong bioluminescence signal was observed to be uniformly localized in the lung lobes, while only a low signal could be detected in the spleen and liver. Quantitative analysis of luciferase protein in lung tissue homogenates demonstrated that aerosol delivery of PPGC NPs significantly increased the production of luciferase protein by nearly 60 times compared with the group treated with nebulized naked m*Luc* solution or PBS (fig. S11C). This finding reveals that PPGC NPs effectively mediate transfection of RNA-based therapeutics in lungs upon nebulization. The hematoxylin and eosin (H&E) staining of both lung and liver tissues demonstrated no toxicity induced by inhaled PPGC NPs (fig. S11D), making it a safe and effective carrier for the pulmonary delivery of RNA-based therapeutics into mouse lungs.

In a murine model of bleomycin-induced pulmonary fibrosis, we investigated the tissue distribution and subcellular localization of Cy5.5-labeled si*IL11*@PPGC NPs upon nebulization by recording Cy5.5 fluorescence signal from organs using IVIS spectrum imaging system. As shown in Fig. 5B, a significantly higher fluorescence signal was observed in the lungs treated with si*IL11*@PPGC NPs when compared with the PBS-treated control group. Quantitative analysis of fluorescence signal demonstrated a uniform distribution of NPs within each lobe (Fig. 5C), indicating that PPGC NPs allowed deep penetration of siRNA therapeutics in the lung tissues. To determine the majority cell subtype colocalized with the si*IL11*@PPGC NPs, lung tissues were digested and cells were analyzed with flow cytometry using markers for epithelial cells (EpCAM), endothelial cells (CD31), and immune cells (CD45), respectively. The results showed that lung epithelial cells were the majority subtype that si*IL11*@PPGC NPs entered, with 34.1% of the total epithelial population displaying Cy5.5 positive, followed by 12% immune cell and 9.89% endothelial cell colocalized with NPs, respectively (Fig. 5, D and E). The gating strategy is presented in fig. S12.

### si*IL11*@PPGC NPs mitigate bleomycin-induced pulmonary fibrosis

**Fig. 6.**
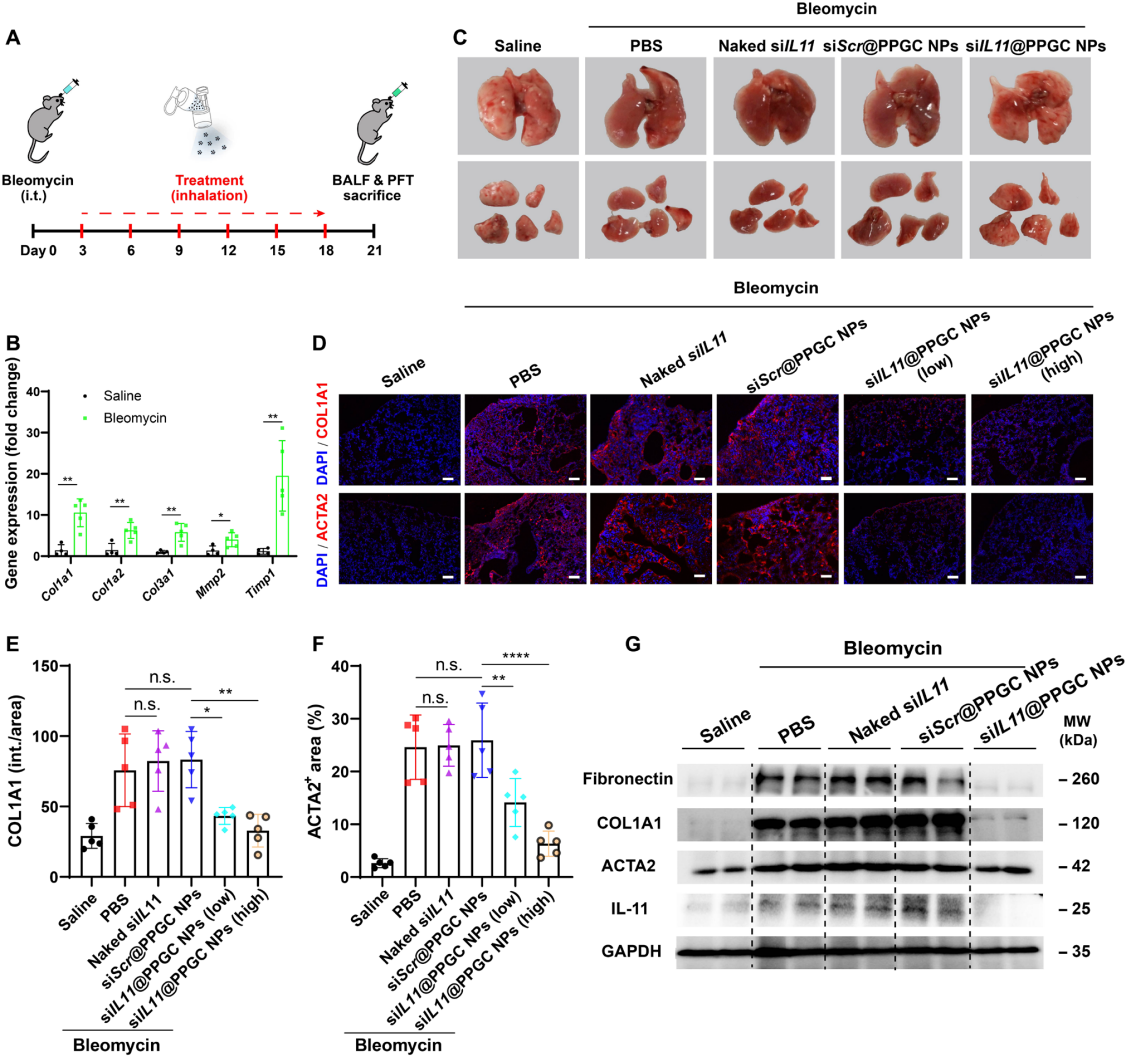
Inhaled si*IL11*@PPGC NPs inhibit fibroblast activation and ECM production in the mouse model of bleomycin-induced pulmonary fibrosis. (**A**) Experimental design of the animal study. i.t., intratracheal. (**B**) Comparison of expression levels of fibrosis- and ECM-related genes in saline and the bleomycin-treated group as determined by qRT-PCR (*N* = 5). (**C**) Representative images of lungs from groups treated with saline, bleomycin + PBS, bleomycin + si*Scr*@PPGC NPs, bleomycin + naked si*IL11*, and bleomycin + si*IL11*@PPGC NPs on day 21 after bleomycin challenge (*N* = 5). (**D**) Representative immunofluorescence images of COL1A1 and ACTA2 from mouse lung sections treated with saline, bleomycin + PBS, bleomycin + si*Scr*@PPGC NPs, bleomycin + naked si*IL11*, bleomycin + si*IL11*@PPGC NPs (0.75 mg/kg of siRNA), and bleomycin + si*IL11*@PPGC NPs (1.5 mg/kg of siRNA). Scale bars, 100 μm. (**E** and **F**) Quantitative analysis of immunofluorescence staining in terms of COL1A1 intensity (E) and ACTA2^+^ area (F) (*N* = 5). (**G**) Western blotting of fibronectin, COL1A1, ACTA2, and IL-11 in mouse lung tissues treated with saline, bleomycin + PBS, bleomycin + si*Scr*@PPGC NPs, bleomycin + naked si*IL11*, and bleomycin + si*IL11*@PPGC NPs. GAPDH was used as a housekeeping standard. Significant differences were assessed using a two-tailed unpaired Student’s *t* test (B) and a one-way ANOVA with Tukey test (E and F). Results are presented as means ± SD. *P < 0.05, **P < 0.01, ****P < 0.0001, n.s., not significant, *P* > 0.05.

To test the impact of si*IL11*@PPGC NPs on lung fibrosis, we established a mouse pulmonary fibrosis model using a single, optimized-dose, intratracheal bleomycin injection in C57BL/6 male mice (fig. S13). Body weight was recorded every day throughout the whole study as a measure of disease burden (fig. S14). The mice were treated with si*IL11*@PPGC NPs via inhalation using a nebulizer every 3 days from days 3 to 21. On day 21, mice were sacrificed for the evaluation of therapeutic outcomes (Fig. 6A). In our preliminary study, mice were nebulized with Cy7-si*IL11*@PPGC NPs at a high dose (1.5 mg/kg of siRNA) and a low dose (0.75 mg/kg of siRNA), and the fluorescence analysis demonstrated a dose-dependent accumulation of NPs in mouse lung tissues (fig. S15). In the following in vivo experiments, si*IL11*@PPGC NPs at doses of both 1.5 and 0.75 mg/kg of siRNA or an equal volume of PBS were used for the evaluation of antifibrotic efficacy.

qRT-PCR result demonstrated that the expression levels of fibrosis-related genes and genes encoded for ECM proteins significantly increased after intratracheal injection of bleomycin (Fig. 6B). The initial histological examination exhibited that groups receiving bleomycin showed hemorrhagic necrosis, which markedly decreased after treatment with the high dose (1.5 mg/kg of siRNA) of si*IL11*@PPGC NPs (Fig. 6C). Immunofluorescence staining images showed that bleomycin treatment induced the expression of ACTA2 and COL1A1 throughout the lung parenchyma, which was remarkably reduced after the aerosol inhalation of si*IL11*@PPGC NPs, especially at a dose of 1.5 mg/kg of siRNA (Fig. 6, D to F). Consistent with the immunofluorescence results, Western blotting demonstrated that inhalation of si*IL11*@PPGC NPs substantially inhibited the production of myofibroblast biomarker ACTA2, ECM proteins fibronectin and COL1A1, as well as IL-11 levels in the lung tissues 21 days after bleomycin challenge, compared with the PBS, si*Scr*@PPGC NPs, or naked si*IL11* control groups (Fig. 6G).

### Histological analysis and signaling pathway evaluation

**Fig. 7.**
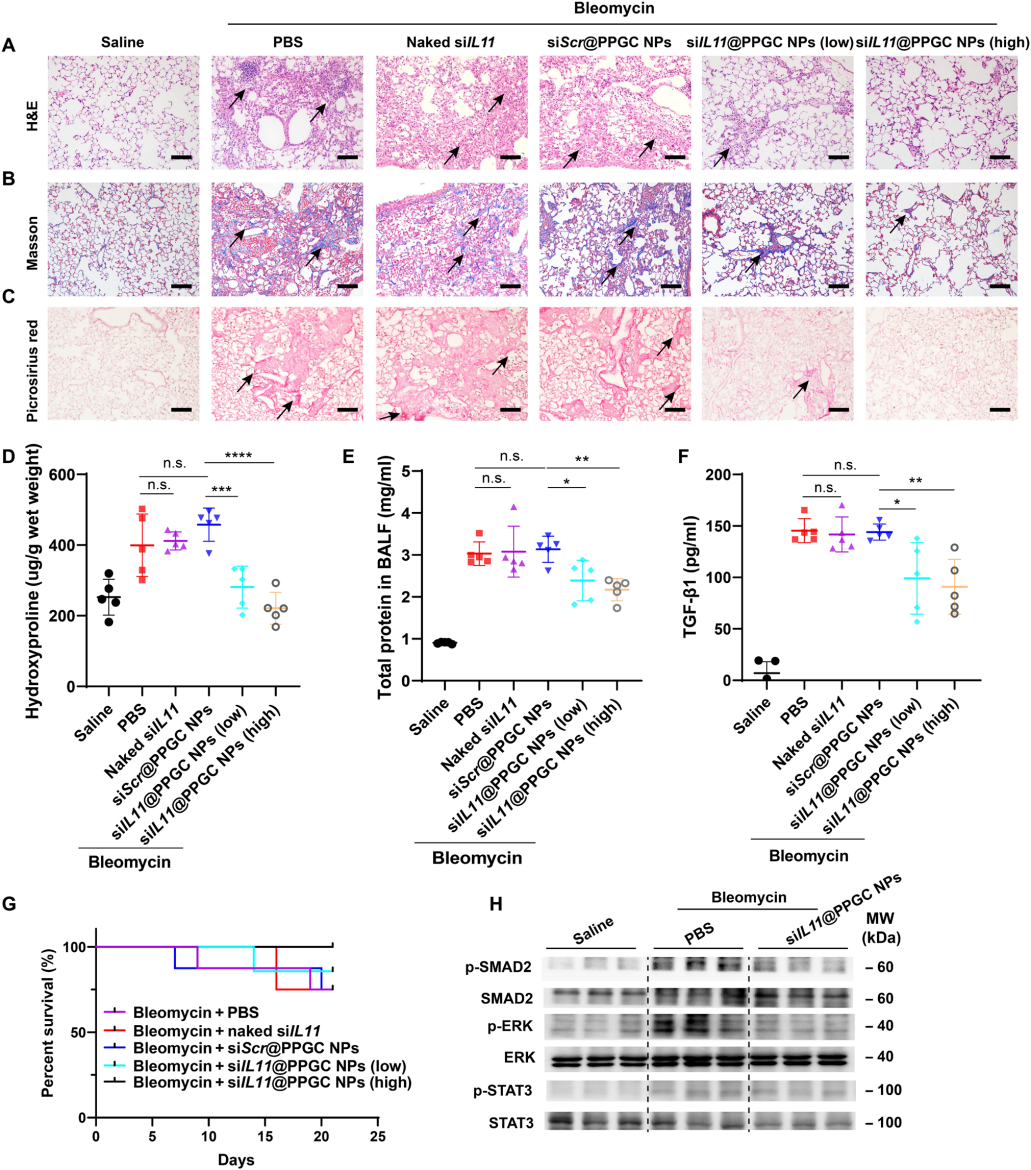
In vivo therapeutic validation of inhaled siRNA therapeutic–mediated IL-11 down-regulation in a pulmonary fibrosis model. (**A** to **C**) Histological analysis of lung sections, including H&E staining (A), Masson’s trichrome staining [muscle fibers and erythrocytes (red), collagen (blue), and nuclei (black-purple)] (B), and Picrosirius red staining (collagen types I and III) (red) (C). Scale bars, 50 μm. (**D**) Measurement of hydroxyproline content in mouse lung tissues (*N* = 5). (**E**) Determination of total protein content in BALF using BCA assay (*N* = 5). (**F**) ELISA quantification of TGF-β1 level in BALF (*N* = 5). (**G**) Survival curve of bleomycin-induced fibrosis animals after treatment with PBS, si*Scr*@PPGC NPs, naked si*IL11*, or si*IL11*@PPGC NPs (*N* = 8). (**H**) Western blotting assay of phosphorylation and total expression of SMAD2, ERK, and STAT3 in lung tissue homogenates collected from different groups. GAPDH was used as a housekeeping standard. Significant differences were assessed using a one-way ANOVA with Tukey test (D to F). Survival analysis was performed using a log-rank test (G). Results are presented as means ± SD. *P < 0.05, **P < 0.01, ***P < 0.001, ****P < 0.0001, n.s., not significant, *P* > 0.05.

The results of H&E staining revealed that si*IL11*@PPGC NP treatment at both low and high doses strongly mitigated the severity of the bleomycin-induced fibrosis by preserving alveolar epithelial structures as compared to the PBS, si*Scr*@PPGC NPs, or naked si*IL11* control groups (Fig. 7A and fig. S16). Masson and picrosirius red staining demonstrated a substantial reduction in collagen deposition and parenchymal disruption after inhalation of si*IL11*@PPGC NPs (Fig. 7, B and C).

Consistent with the histological analysis, lung hydroxyproline content was greatly reduced by si*IL11*@PPGC NP treatment, especially at the dose of 1.5 mg/kg of siRNA (Fig. 7D). Bleomycin challenge resulted in a marked increase in the hydroxyproline content up to 410 μg/g of wet lung tissues, which was decreased to 200 μg/g of wet lung tissues by si*IL11*@PPGC NP treatment at a dose of 1.5 mg/kg of siRNA, nearly back to the level of healthy controls. Total protein content in the bronchoalveolar lavage fluid (BALF) markedly escalated after bleomycin challenge, and si*IL11*@PPGC NP treatment significantly reversed the bleomycin-induced increase in terms of total protein content in BALF (Fig. 7E). Considering that TGF-β1 is a critical profibrotic mediator in fibrosis development and involved in diverse pathological processes such as cell apoptosis, myofibroblast differentiation, and collagen deposition (*43*–*46*), TGF-β1 in BALF collected from the group treated with si*IL11*@PPGC NPs and control groups treated with PBS, si*Scr*@PPGC NPs, or naked si*IL11* was also determined by enzyme-linked immunosorbent assay (ELISA). Figure 7F shows that si*IL11*@PPGC NP treatment significantly inhibited the bleomycin-induced increase of TGF-β1, which is highly relevant to the dose of the inhaled NPs.

As shown in Fig. 7G, two of the eight bleomycin-induced fibrosis mice died in the early period in the PBS-treated group. In contrast, all mice stayed alive after treatment with si*IL11*@PPGC NPs at a dose of 1.5 mg/kg of siRNA during the entire experimental period, indicating that si*IL11*@PPGC NPs improved the overall survival of bleomycin-induced fibrosis mice. Blood chemistry including serum alanine transaminase (ALT) and aspartate transaminase (AST) was examined, and the results showed no toxicity for si*IL11*@PPGC NP treatment groups compared with the healthy controls (fig. S17, A and B). In addition, nebulization of si*IL11*@PPGC NPs did not induce any significant changes in the organ coefficient of fibrosis animals compared with the healthy controls, making them a safe vector for inhalable siRNA therapeutics (fig. S17C).

To investigate the signaling pathways pivotal for IL-11–driven pulmonary fibrosis, we assessed the activation status of SMAD2, ERK, and STAT3 by Western blotting of lung homogenates from the PBS-treated group, si*IL11*@PPGC NP–treated group, or healthy controls. As shown in Fig. 7H, after treatment of inhaled si*IL11*@PPGC NPs, there was an obvious inhibition in expression of p-SMAD2 and p-ERK in lungs, but p-STAT3 remained elevated. ERK- and SMAD2-dependent myofibroblast differentiation and ECM deposition are critically involved in the IPF disease pathology. The in vivo results suggested that the aforementioned antifibrotic effects mediated by the inhaled si*IL11*@PPGC NP treatment could be attributed to the effective blocking of IL-11–induced ERK and SMAD2 activation. Overall, the developed inhalable si*IL11*@PPGC NPs target the downstream *IL11* and avoid the adverse effects associated with inhibition of TGF-β1 directly or indirectly (*47*, *48*), offering a safe, noninvasive, and potent therapeutic approach for IPF treatment.

### Inhaled si*IL11*@PPGC NP treatment improves pulmonary function

**Fig. 8.**
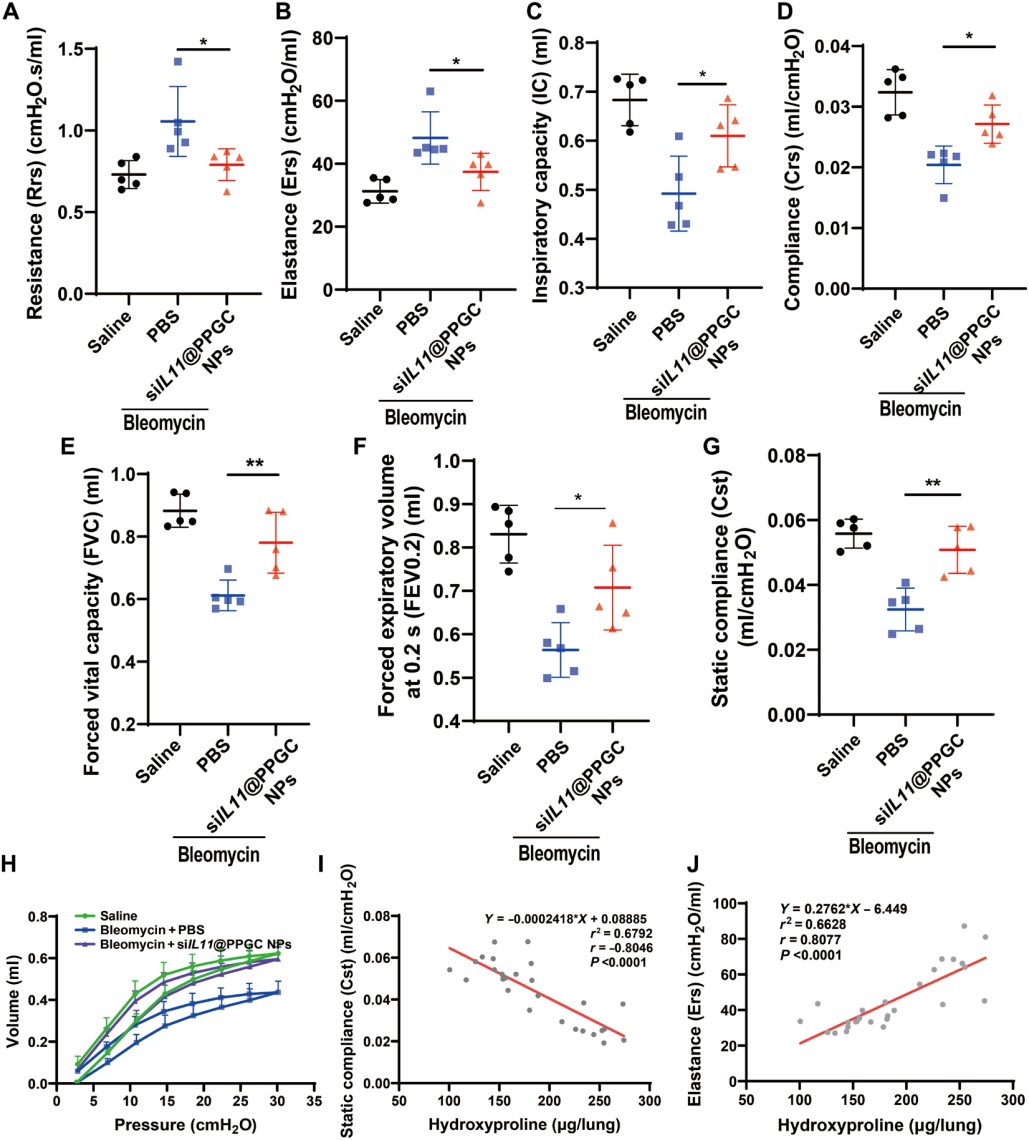
Inhalation of si*IL11*@PPGC NPs restores the pulmonary function in the mouse model of bleomycin-induced pulmonary fibrosis. (**A** to **G**) Measurements of pulmonary function parameters including resistance (Rrs) (A), elastance (Ers) (B), inspiratory capacity (IC) (C), compliance (Crs) (D), forced vital capacity (FVC) (E) and forced expiratory volume at 0.2 s (FEV0.2) (F), and static compliance (Cst) (G) (*N* = 5). (**H**) Measurements of PV loop (*N* = 5). (**I** and **J**) Correlation analysis between hydroxyproline content and static compliance (Cst) (I) as well as elastance (Ers) (J) (*N* = 27). Significant differences were assessed using a one-way ANOVA with Tukey test (A to G). Correlation coefficient (*r*) was assessed using a nonparametric Spearman correlation analysis (I and J). Results are presented as means ± SD. *P < 0.05, **P < 0.01.

Last, we sought to predict the clinical impact of si*IL11*@PPGC NP treatment on impaired lung function using the forced oscillation technique. The pulmonary function test (PFT) was performed on day 21 in both mouse models of pulmonary fibrosis and healthy controls to assess the therapeutic impact of si*IL11*@PPGC NPs on lung function. Respiratory resistance (Rrs) and elastance (Ers) were two important parameters that severely increased in the experimental murine fibrosis models, indicating an impaired constriction level and elastic stiffness in the bleomycin-injured lungs (Fig. 8, A and B). Intriguingly, they were both significantly improved after si*IL11*@PPGC NP treatment.

Other pulmonary function parameters including inspiratory capacity (IC; normalized to body weight), compliance (Crs), forced vital capacity (FVC), forced expiratory volume at 0.2 s (FEV0.2), and static compliance (Cst) showed decline following bleomycin-induced injury compared with healthy controls (Fig. 8, C to G). As expected, the above parameters were all significantly rescued by si*IL11*@PPGC NP treatment, especially for FVC and Cst.

The pressure-volume (PV) loop of mice showed a characteristic downward shift after bleomycin challenge, suggesting increased lung stiffness. The inhalation of si*IL11*@PPGC NPs restored the PV loop nearly back to the level of healthy controls, indicative of pulmonary function recovery (Fig. 8H).

We then investigated the relationship of pulmonary function parameters and hydroxyproline content, a major indicator of collagen content in the fibrotic tissue, by establishing a linear regression model. Figure 8I illustrates a strong negative correlation between static compliance and hydroxyproline content. Besides, the elastance was positively related to the hydroxyproline content (Fig. 8J). The correlations between hydroxyproline content and other pulmonary function parameters were also shown in fig. S18. Together, we demonstrate that the therapeutic inhibition of IL-11 using the inhalable si*IL11*@PPGC NPs holds great promise in resolving pulmonary fibrosis and restoring healthy lung function.

## DISCUSSION

The exact etiology for the development of IPF is unknown and needs to be further elucidated, but virtually all fibrotic processes are associated with persistent FMT, migration, and excessive deposition of ECM proteins in the distal lung, which irreversibly lead to the disruption of lung architecture, decline of lung function, and, ultimately, death. Recent studies have revealed the prominent role of IL-11 in driving ERK-dependent myofibroblast differentiation and ECM production that are involved in IPF pathology. These findings, combined with our immunohistochemistry staining result, prioritize IL-11 as a promising target, and effective blockage of IL-11 signaling pathway may open up a new avenue for IPF therapy.

RNAi therapeutics such as siRNA can be rationally designed to specifically silence the expression of target genes that are considered “undruggable” and/or require complex and time-consuming development of effective inhibitors, thus holding great promise for revolutionizing the treatment of human diseases (*49*, *50*). Since the approval of the first lipid nanoparticle (LNP)–assisted siRNA therapeutics (patisiran) for the treatment of hATTR amyloidosis, three other siRNA therapeutics that are chemically modified with *N*-acetylgalactosamine (GalNAc) have been successfully developed for hepatocyte-targeting delivery by specifically binding with asialoglycoprotein receptor (ASGPR) on the cell surface (*51*). Despite the enormous progress that has been made in the delivery of oligonucleotide therapeutics in vivo, it remains challenging to achieve targeted delivery to organs and tissues other than the liver, such as the lung.

The combination of localized administration routes and nanoparticulate formulations may be considered as a potential approach to overcome the problems caused by the various biological barriers encountered in gene delivery (*52*). The noninvasive aerosol inhalation route is one of the most preferable modes of local drug administration for lung disorders, which has many unique advantages in addition to painlessness and relative convenience, such as increased drug deposition throughout the entire bronchiolar and alveolar epithelium, low systemic risk, improved patient compliance and adherence, feasibility for repeated procedures, and rapid onset of pharmacologic action. Direct delivery of siRNA therapeutics to the lung via aerosol inhalation of nanoparticulate formulations may provide a promising and clinically relevant therapeutic approach for the treatment of pulmonary diseases. Here, we developed a unique lipid-polymer NP platform consisting of lipid-like compound G0-C14 and PLGA-PEG to address the barriers associated with aerosol delivery of siRNA against *IL11*, a point of signaling convergence downstream of multiple profibrotic stimuli. The developed NPs, termed si*IL11*@PPGC NPs, enabled pulmonary delivery of siRNA and were capable of depositing si*IL11* through the whole bronchiolar and alveolar epithelium after nebulization. si*IL11*@PPGC NPs have good biocompatibility and can withstand the drastic shearing forces generated during nebulization and efficiently silence the target gene. In vitro studies demonstrated that the locally delivered si*IL11*@PPGC NPs effectively inhibited ECM deposition and differentiation and migration in fibroblasts by blocking the IL-11 signaling pathway. In addition, nebulization of the NPs to mice did not elicit any observable local immune reaction in the lung, liver, or any aberrant cellular or tissue alterations. All these features are essential for the development of siRNA delivery systems suitable for inhaled RNA therapy.

The bleomycin-induced pulmonary fibrosis model is the most clinically relevant and has been widely used for the study of IPF (*53*, *54*). In the experimental mouse model of bleomycin-induced pulmonary fibrosis, significant alveolar epithelial damage and ECM deposition were observed following bleomycin treatment. Using this mouse model, we found that aerosol inhalation of si*IL11*@PPGC NPs resulted in a significant decline in the production of myofibroblast biomarker ACTA2, ECM proteins fibronectin and COL1A1, IL-11, and hydroxyproline content in the lung tissues. Histological analysis of the lung sections also demonstrated that NP treatment substantially attenuated bleomycin-induced thickened alveolar septa, restored the structure of impaired alveolar barrier, and reduced collagen deposition and parenchymal disruption. The NP-mediated amelioration of pulmonary fibrosis was associated with suppression of TGF-β1/SMAD2 and IL-11/ERK signaling pathways as evidenced by Western blotting analysis. In addition, inhalation of si*IL11*@PPGC NPs remarkably enhanced recovery of pulmonary function from bleomycin-induced lung injury, with improved overall survival.

Although the results are encouraging, there is room for improvement regarding the present work. First, the current study is limited to the bleomycin-induced pulmonary fibrosis mouse model that may not reflect all pathologic features of IPF. Additional investigations in various animal models (e.g., silica-induced pulmonary fibrosis model and humanized severe combined immunodeficient mouse model of IPF) and different animal species (e.g., nonhuman primate) would help further validate the safety and efficacy of the inhalable si*IL11*@PPGC NP–based approach for potential translation (*18*). Second, it has been reported that siRNA could trigger interferon (IFN) response and induce the expression of IFN-stimulated genes after they are recognized by double-stranded RNA (dsRNA) sensors such as Toll-like receptors (TLRs), dsRNA-dependent protein kinase R (PKR), and retinoic acid–inducible gene I (RIG-1) (*55*). On the other hand, some cationic NPs may evoke an immunological response when interacting with TLR2 and TLR4 located on the cell surfaces of macrophages and other cells, leading to the secretion of cytokines and chemokines including IL-1α, IL-1β, IL-10, IL-6, and tumor necrosis factor–α (TNF-α) (*56*). Given that chronic diseases such as IPF generally require a long period of treatment, it is necessary to systemically assess whether the inhaled si*IL11*@PPGC NPs can induce any adverse immune effects, as well as the impact of RNA sequence, NP properties, administration route, and preexisting immune status of model animals on the induced immunotoxicity. In addition, we will use NPs loaded with chemically modified siRNA to further enhance potency and specificity, decrease off-target–induced toxicity, and reduce dosing frequency. Third, future studies will focus on developing a robust and scalable manufacturing process of such inhalable nanoparticulate siRNA therapeutics.

This NP platform can be applied for simultaneous pulmonary delivery of multiple siRNAs to target different profibrogenic pathways, which should provide synergistic benefits and further improve the antifibrotic effects. Since cancer-associated fibrosis is a critical component of the tumor microenvironment, si*IL11*@PPGC NPs can be used as a “lead drug” to resolve fibrotic lesions so as to enhance the penetration of anticancer drugs, which may provide insights into exploiting and validating new treatments and targets for lung cancers. In summary, we present a versatile NP platform with mucus penetration property and investigated its therapeutic efficacy for aerosol delivery of siRNA therapeutics. The inhalable nanoparticulate siRNA has the unique advantages of noninvasiveness, feasibility of repeated procedures, low systemic toxicity, accessibility to the distal lung, and improved compliance. This work exhibits promising translational relevance in the siRNA therapeutic–based treatment for IPF and other lung diseases including lung cancer, asthma, and COVID-19–associated pneumonia.

## MATERIALS AND METHODS

### Experimental design

The aim of this study was to develop inhaled siRNA NPs that can silence the expression of profibrogenic IL-11, which is significantly involved in IPF pathology, and to validate the therapeutic effect of this strategy in bleomycin-induced pulmonary fibrosis. The physicochemical properties were characterized by measuring stability, size, zeta potential, morphology, cell internalization, cell viability, silence efficiency, and transmucosal capacity. In vitro experiments in MLFs were used to verify the effect of NPs on inhibiting cell migration, myofibroblast differentiation, and ECM deposition, and to clarify the downstream signaling pathway of IL-11. All the cell culture experiments were replicated independently at least twice per experiment. In vivo animal studies were performed in mice with bleomycin-induced pulmonary fibrosis to determine the therapeutic efficacy of NPs, including immunofluorescence staining, histology analysis, hydroxyproline and BALF TGF-β1 content, Western blotting, and PFT. All the animal experiments were carried out according to the protocols approved by the Institutional Animal Care and Use Committee at Shanghai Jiao Tong University. Mice were randomly assigned to experimental groups on the day before treatment.

### Materials

Poly(d,l-lactide-*co*-glycolide) (50:50; 0.55 to 0.75 dl/g) was purchased from Lactel Absorbable Polymers. Heterobifunctional PEG polymers NH_2_-PEG-OCH_3_ (molecular weight of 3.4 kDa) were purchased from Layson Bio. 1-Ethyl-3-[3-dimethylaminopropyl] carbodiimide (EDC) hydrochloride, *N*-hydroxysuccinimide (NHS), and *N*,*N*-diisopropylethylamine (DIPEA) were purchased from Beijing Yinuokai. PAMAM dendrimers of generation 0 (G0), 1,2-epoxytetradecane (C14), polyvinyl alcohol (PVA; molecular weight = 13,000 to 23,000 kDa, 87 to 89% hydrolyzed), and *N*,*N*-dimethylformamide (DMF) were purchased from Sigma-Aldrich. The recombinant protein TGF-β1 and TGF-β1 ELISA kits were purchased from PeproTech and Neobioscience, respectively. The hydroxyproline assay kit was obtained from Nanjing Jiancheng, and d-luciferin was from MaoKang Biological Technology.

The antibodies used in this work are described as follows: anti-ACTA2 (1:3000 for Western blotting, Abcam, UK), anti-ACTA2 (1:1000 for immunohistochemistry and 1:300 for immunofluorescence, Servicebio, China), anti–IL-11 (1:1000 for Western blotting and immunohistochemistry, Proteintech, China), horseradish peroxidase (HRP)–conjugated goat anti-mouse IgG (1:5000 for Western blotting, Proteintech, China), HRP-conjugated goat anti-rabbit immunoglobulin G (IgG) (1:30000 for Western blotting, Signalway Antibody, USA), anti-COL1A1 (1:1000 for Western blotting and 1:300 for immunofluorescence, Servicebio, China), anti-fibronectin (1:1000 for Western blotting, Servicebio, China), anti–glyceraldehyde-3-phosphate dehydrogenase (GAPDH) (1:1000 for Western blotting, Servicebio, China), Alexa Fluor 488–conjugated goat anti-rabbit IgG (1:500 for immunofluorescence, Servicebio, China), Cy5-conjugated goat anti-rabbit IgG (1:500 for immunofluorescence, Servicebio, China), anti-ERK1/2 (1:1000 for Western blotting, Cell Signaling Technology, USA), anti–p-ERK1/2 (1:2000 for Western blotting, Cell Signaling Technology, USA), anti-SMAD2 (1:1000 for Western blotting, Cell Signaling Technology, USA), anti–p-SMAD2 (1:1000 for Western blotting, Cell Signaling Technology, USA), anti-STAT3 (1:1000 for Western blotting, Servicebio, China), and anti–p-STAT3 (1:2000 for Western blotting, Cell Signaling Technology, USA).

Schematic illustration of the nebulizer apparatus is provided in Fig. 5A. Briefly, the inhalation chamber is connected with a custom-made nose cone that is designed in such a way that only the noses of the mice are exposed to the aerosol cloud. The upstream side of the inhalation chamber is connected to a nebulizer (Aerogen, Ireland), where doses are added to produce aerosols through a vibrating mesh. The downstream side is connected to a vacuum pump to maintain continuous aerosol flow in this system. The pressure manometer that is connected to the chamber is constantly monitored, and the valves are adjusted to maintain atmospheric pressure in the chamber.

### Synthesis of PLGA-PEG and G0-C14

Copolymer PLGA-PEG and Cy5.5-labeled PLGA-PEG (PLGA-PEG-Cy5.5) were synthesized as described previously (*39*, *57*). Briefly, carboxy-terminated PLGA was first activated with EDC and NHS followed by precipitation in precooled methanol/diethyl ether (50:50, v/v) two times. The obtained PLGA-NHS was then reacted with NH_2_-PEG-OCH_3_ or NH_2_-PEG-Cy5.5 in the presence of DIPEA followed by removal of the residual organic solvent. The yielded PLGA-PEG was characterized by ^1^H NMR (CDCl_3_, 400 MHz, Agilent, USA) as shown in fig. S3.

G0-C14 was synthesized by reacting 1,2-epoxytetradecane with generation 0 of ethylenediamine core-PAMAM dendrimer according to a previously described procedure (*39*) but with significant modification and optimization in ratios of reagents used in the G0-C14 synthesis. In brief, PAMAM dendrimer and 1,2-epoxytetradecane were mixed at a molar ratio of 1:5 and stirred at 90°C vigorously for 2 days, yielding a final product with three less lipid tail than the total possible for a designated amine monomer. The ^1^H NMR (CDCl_3_, 400 MHz, Agilent, USA) spectrum of G0-C14 is shown in fig. S4.

### siRNA complexation ability of G0-C14

The siRNA complexation ability of G0-C14 was assessed by electrophoresis. siRNA was complexed with G0-C14 at various weight ratios ranging from 1 to 40 followed by incubation at room temperature for 30 min. The samples were then mixed with loading dye (Beyotime), and electrophoresis was run in 1% agarose gel for 10 min at 110 V. The double-strand marker A (Sangon Biotech) was used as a ladder. Last, the gel was imaged using the ChemiDoc system (Bio-Rad, USA), and the bands were analyzed.

### Preparation of PPGC NPs

PPGC NPs were prepared through self-assembly of PLGA-PEG and the cationic G0-C14 using a nanoprecipitation method. PLGA-PEG and G0-C14 were dissolved in DMF, and the yielded mixture solution was added to siRNA aqueous solution at a weight ratio of 30:30:1 (PLGA-PEG:G0-C14:siRNA). This solution was then quickly nanoprecipitated into 10 ml of 0.25% PVA aqueous solution and stirred at room temperature for 10 min to enable the stabilization. The NPs were washed three times in double-distilled water and concentrated to an appropriate volume using Amicon tubes with a molecular weight cutoff of 100 kDa. For Cy5.5-labeled si*IL11*@PPGC NPs used in the biodistribution study and m*Luc*@PPGC NPs used in the transfection study, 10% (w/w) of total PLGA-PEG was replaced with PLGA-PEG-Cy5.5 to prepare the Cy5.5-labeled PPGC NPs. The solvents used in the NP preparation were all deoxyribonuclease (DNase)/RNase free.

The yielded NPs were nebulized into aerosol droplets using an AeroNeb vibrating mesh nebulizer (Aerogen, Ireland). The nebulized NP aerosol was then collected for TEM imaging and measurements of DLS, siRNA release, and encapsulation efficiency.

### Isolation of primary MLFs

Primary MLFs were isolated from the lungs of 8-week-old male C57BL/6 mice. The lung was excised, minced, and digested in serum-free Dulbecco’s modified Eagle’s medium (DMEM) containing collagenase I (1 mg/ml) and 1% penicillin-streptomycin for 30 min at 37°C. Lung tissues were then neutralized with complete DMEM containing 10% FBS and centrifuged at 1700 rpm for 3 min. The obtained tissue pellet was washed with PBS three times and finally resuspended in complete DMEM. MLFs were allowed to explant from tissues for 4 days, and cells were detached with 0.25% trypsin-EDTA and passaged at a ratio of 1:3. MLFs used for downstream experiments were between passages 3 and 5. The purification of primary lung fibroblasts could be achieved through the differential adhesion method. To enable the myofibroblast differentiation, MLFs were pretreated with TGF-β1 at a concentration of 10 ng/ml for 24 hours. Both immunofluorescence and Western blotting were applied to measure the expression of ACTA2, a biomarker of the myofibroblasts, to characterize the differentiation.

### PPGC NP stability assessment and physicochemical characterization

The stability of naked siRNA and NP-encapsulating siRNA against RNase were tested and visualized with agarose gel electrophoresis. Briefly, naked siRNA or siRNA@PPGC NPs were incubated with RNase (10 μg/ml) at 37°C for 0, 15, 30, 60, 120, or 240 min. The NPs were then collected by centrifugation at 12,000 rpm for 10 min. The pellet was dissolved in chloroform, and the siRNA was extracted in 0.5 M NaCl containing 0.1% SDS (*58*). Electrophoresis was performed on GelRed-infused 3% agarose gels followed by imaging under ultraviolet (UV) light.

To check the stability of PPGC NPs in serum, NPs were incubated in PBS containing 10% FBS at pH 7.4 or 6.8 at 37°C for 24 hours. At predetermined time points, an aliquot of NP solution was taken for particle size measurements using DLS (Malvern, UK).

The morphologies of PPGC NPs before and after nebulization were characterized using TEM (Thermo Fisher Scientific, USA). The size and zeta potential of NPs were measured using DLS.

### Determination of release profile and encapsulation efficiency

To determine the release kinetics, FAM-labeled siRNA (FAM-siRNA) was encapsulated into the PPGC NPs to obtain FAM-siRNA@PPGC NPs. A suspension of NPs in PBS was dialyzed against PBS (pH 7.4 and pH 5.0) in Float-A-Lyzer (molecular weight = 300 kDa) at 37°C with gentle stirring. At predetermined time points, a certain volume of PBS was removed and an equal volume of PBS was added into the inner tubes. A standard curve correlating fluorescence and siRNA concentration was used to determine the amount of siRNA released from the NPs. The fluorescence intensity was measured with a multimode microplate reader (excitation/emission, 494/522 nm; Tecan, Switzerland).

The encapsulation efficiency (EE) was determined with the Quant-iT RiboGreen reagent according to the instructions. Briefly, NPs loaded with siRNA were prepared and nebulized according to the aforementioned method. A 3-μl volume of the NP solution was mixed with 117 μl of 1× TE (Tris-EDTA) buffer (A) or 2% Triton X-100 (B) and vortexed for 2 min. Free siRNA standard was diluted in a series of concentrations. NP samples and siRNA standard were incubated with an equal volume of 1:200 diluted RiboGreen reagent for 5 min. The fluorescence intensity was determined with a microplate reader (excitation/emission, 480/520 nm; Tecan, Switzerland). Encapsulation efficiency (EE %) was calculated using the following formula

EE (%)=(fluorescence of B–fluorescence of A)/(fluorescence of B)×100%

### Cell uptake activity

FAM-siRNA was encapsulated into PPGC NPs according to the aforementioned NP preparation method. The obtained FAM-siRNA@PPGC NPs (before or after nebulization) were incubated with MLFs for 4 hours. MLFs were detached, washed with PBS, and measured by flow cytometry to quantify the percentage of FAM-positive MLFs.

LSCM (Leica, Germany) was used to visualize the cellular internalization of NPs in MLFs and A549. Briefly, cells were incubated with Cy5.5-labeled NPs for 4 hours and fixed in 4% paraformaldehyde. Cells were stained with 4′,6-diamidino-2-phenylindole (DAPI) for the visualization of nuclei. TGF-β1–induced myofibroblasts were also stained with primary antibody of ACTA2 followed by incubation with Alexa Fluor 488–conjugated secondary antibody.

### Cell viability assay

MLFs were seeded at a density of 1 × 10^4^ cells per well in a 96-well plate overnight and incubated with different concentrations of PPGC NPs for 24 hours. Cells without NP treatment were used as a control. Then, the medium was discarded and serum-free medium containing 10 μl of CCK8 solution was added into each well, followed by incubation at 37°C for 2 hours. The absorbance at 450 nm was measured using a multimodal plate reader (Tecan, Switzerland).

### Cellular uptake mechanism

MLFs were seeded at a density of 5 × 10^4^ cells per well in a 24-well plate overnight at 37°C in 5% CO_2_. The cells were then pretreated with CPZ, EIPA, or filipin (alone and in combination) for 30 min to block clathrin-mediated endocytosis, micropinocytosis, and caveolae-mediated endocytosis. The MLFs were then incubated with FAM-siRNA@PPGC NPs for 4 hours. The medium containing NPs was then discarded, and cells were harvested to check the internalization with flow cytometry.

### Silencing efficiency of si*IL11*

A series of siRNAs targeting different regions of *IL11* mRNA were synthesized by GenePharma. The sequences of si*IL11*s are summarized in table S1. The synthesized si*IL11*s were encapsulated into PPGC NPs, and the yielded si*IL11*@PPGC NPs were incubated with MLFs for 4 hours. The total RNA was extracted with TRIzol 24 hours later. qRT-PCR was used to measure the *IL11* mRNA level. The qPCR primers of *IL11* mRNA are as follows: 5′-TGTTCTCCTAACCCGATCCCT-3′ (forward) and 5′-CAGGAAGCTGCAAAGATCCCA-3′ (reverse).

### In vitro penetration assay using artificial mucus model

One milliliter of 10% (w/v) gelatin solution was added into a 24-well plate and hardened at room temperature. Briefly, artificial mucus was prepared by adding 500 mg of DNA, 250 mg of mucin, 250 μl of sterile egg yolk emulsion, 0.295 mg of diethylenetriamine pentaacetic acid, 250 mg of NaCl, 110 mg of KCl, and 1 ml of RPMI 1640 into 50 ml of water. The mixture was stirred overnight until a homogeneous dispersion was obtained. In vitro model of penetration was constructed by adding 1 ml of artificial mucus to the surface of the gelatin layer.

FAM-siRNA was encapsulated into PPGC NPs to yield FAM-siRNA@PPGC NPs with PEG coating according to the aforementioned nanoprecipitation method. We then encapsulated FAM-siRNA into NPs consisting of PLGA and G0-C14 by nanoprecipitation to yield FAM-siRNA@PGC NPs without PEG coating while keeping other components as a control. The resulting NPs (with or without PEG coating) at equivalent dose of FAM-siRNA were added to the artificial mucus. At predetermined time points, the artificial mucus layer was withdrawn and the fluorescence signal of FAM-siRNA@NPs penetrating into the gelatin was quantified using a multimodal plate reader (Tecan, Switzerland).

### In vivo transmucosal penetration study

The impact of PEG coating on the transmucosal delivery property of NPs was also investigated in vivo. FAM-siRNA@PGC NPs without PEG coating and Cy7-siRNA@PPGC NPs that have PEG coating were prepared as described above. One hundred microliters of mixture of both NPs with and without PEG coating was administered to the mouse lung via intratracheal injection. Thirty minutes after administration, mice were sacrificed and lungs were collected, fixed in 4% paraformaldehyde, and embedded in optimal cutting temperature (OCT). Lungs were sectioned at 10-μm intervals on a Cryostat (Thermo Fisher Scientific, USA) and stained with DAPI before using Prolong Gold antifade reagent. The fluorescence images were acquired using the LSCM (Leica, Germany). For each mouse (*N* = 3), at least one section was acquired at the z thickness of 100 to 120 μm, and three randomly selected zones from the section were imaged for a total of nine measurements. ImageJ software was used to quantify the percent areas of positive fluorescence signal from Cy7-siRNA@PPGC NPs and FAM-siRNA@PGC NPs in lung tissue sections.

### Immunofluorescence staining and microscopy

MLFs were seeded on coverslips in 12-well plates at a density of 1.2 × 10^5^ cells per well and allowed to attach in growth medium at 37°C in a 5% CO_2_ incubator overnight. Cells were then incubated with si*IL11*@PPGC NPs or si*Scr*@PPGC NPs in serum-free DMEM for 4 hours and further incubated in complete medium for another 20 hours. The MLFs were then starved in serum-free DMEM for 24 hours. The starved cells were subsequently cultured in DMEM containing TGF-β1 (10 ng/ml) and 1% FBS for an additional 24 hours. After TGF-β1 stimulation, cells were fixed in 4% paraformaldehyde and permeabilized with 0.1% Triton X-100. Cells were then blocked with 3% bovine serum albumin (BSA) before incubation with primary antibodies against ACTA2 and COL1A1, and subsequently visualized using Alexa Fluor 488–conjugated secondary antibodies. The cell nuclei were counterstained with DAPI. The images were captured with a fluorescence microscope (Olympus, Japan), and the percentage of ACTA2^+^ fibroblasts and the mean fluorescence intensity of COL1A1 staining were analyzed and quantified with ImageJ software.

### Wound-healing assay

MLFs were seeded at a density of 1.2 × 10^5^ cells per well and cultured in DMEM containing 10% FBS and 1% penicillin-streptomycin reaching 90% confluence. Cells were incubated with si*Scr*@PPGC NPs, si*IL11*@PPGC NPs, and an equal volume of PBS for 4 hours, followed by incubation in complete medium at 37°C for another 24 hours. The cell monolayer was scraped straightly with a sterile 200-μl pipette and washed with PBS three times to remove the cell debris and detached cells. After incubation in serum-free DMEM for 24 hours, the migration images were collected to determine the healing percentage.

### Migration assay

The motility of MLFs was assessed using 24-well Boyden chamber migration assay. Briefly, equal numbers of MLFs were seeded overnight and then treated with si*Scr*@PPGC NPs or si*IL11*@PPGC NPs for 4 hours. Cells were washed and further cultured in complete medium for another 20 hours. After starving in serum-free DMEM for 24 hours, MLFs were allowed to migrate toward TGF-β1, which was used as a chemoattractant. After 24 hours of incubation at 37°C, nonmigratory cells on the upper side of the membrane were removed with cotton swabs, and migratory cells were fixed in 4% paraformaldehyde and stained with cell staining solutions. Images were collected, and the number of the migratory cells from two nonoverlapping fields of each membrane was counted under ×20 magnification.

### Animals

Eight- to 10-week-old male C57BL6 mice were purchased from Beijing Vital River. Mice were anesthetized, and a single intratracheal injection of bleomycin sulfate at a dose of 1.5 U/kg was used to establish the experimental pulmonary fibrosis model. For in vivo therapeutic studies, PBS, si*Scr*@PPGC NPs, naked si*IL11*, and si*IL11*@PPGC NPs containing 0.75 or 1.5 mg/kg of siRNA were given via inhalation route on days 3, 6, 9, 12, 15, and 18 after bleomycin insult using the inhalation device. Lungs were harvested on day 21 after bleomycin challenge. All the procedures were approved by the Institutional Animal Care and Use Committee at Shanghai Jiao Tong University.

### IVIS spectrum imaging analysis

Equivalent doses of m*Luc*@PPGC NPs or naked m*Luc* solution were loaded into the nebulizer, and an air flow was used to direct the aerosol along the spacer into the chamber until no more aerosol could be observed. Mice treated with PBS were used as negative control. Twenty-four hours after nebulization, mice were intraperitoneally injected with luciferin (0.15 mg/g). Ten minutes later, mice were sacrificed and organs were harvested and imaged on an IVIS system (PerkinElmer, USA).

### Biodistribution study

Fluorescently labeled si*IL11*@PPGC NPs used in this study were prepared by encapsulating si*IL11* into Cy5.5-labeled PPGC NPs as described above. Twenty-four hours after inhalation of the Cy5.5-labeled si*IL11*@PPGC NPs, bleomycin-induced fibrosis mice were sacrificed and organs were harvested and imaged on an IVIS system (PerkinElmer, USA). To determine the majority cell subtype colocalized with the si*IL11*@Cy5.5-PPGC NPs, lungs were collected, minced, and digested in PBS containing collagenase I (201.3 U/ml; Yeason), 0.92 M Hepes (Beyotime), and DNase I (50.3 U/ml; Sigma-Aldrich) at 37°C for 1 hour. The obtained digestion was filtered using a 70-μm cell strainer and processed using red blood lysis buffer for 5 min. The samples were then centrifuged at 400*g*, resuspended in PBS containing 0.5% BSA, and filtered through a 40-μm cell strainer. The samples were subsequently incubated with antibodies against epithelial (EpCAM–Alexa Fluor 647), immune (CD45-phycoerythrin), and endothelial (CD31–Brilliant Violet 421) cell markers at 4°C for 30 min. Flow cytometry (BD Biosciences, USA) was used to determine the internalization of NPs in various cell subtypes.

### Determination of TGF-β1 level in BALF

The mice were anesthetized with pentobarbital (50 mg/kg, intraperitoneally) and placed in the dorsal decubitus position. Scissors were used to make a small incision in the neck skin. Salivary glands and sternohyoid muscles were separated to expose the trachea. A cotton thread was placed under the trachea, and a small semi-incision was made to allow the insertion of a 21-gauge lavage tube, which was further stabilized with the cotton thread. Subsequently, the lungs were lavaged two times with 1-ml sterile saline-EDTA (*46*), and the BALF was collected and combined. The total protein content was measured using a bicinchoninic acid (BCA) protein assay kit. The TGF-β1 level in BALF was determined using an ELISA kit.

### Determination of hydroxyproline content in mouse lungs

The hydroxyproline content in mouse lungs was determined using an alkaline hydrolysis kit according to the manufacturer’s instructions. Briefly, 30- to 100-mg lung tissues were accurately weighed and hydrolyzed in alkaline solution at 100°C for 20 min. The hydrolysate was adjusted to a pH of 6.0 to 6.8, treated with activated carbon, and centrifuged at 3500 rpm for 10 min. The collected supernatant was incubated with detection agents at 60°C for 15 min, and absorbance at 550 nm was measured to determine the hydroxyproline content using a UV spectrophotometer (SHIMADZU, Japan).

### Histology analysis

For the mouse model of bleomycin-induced pulmonary fibrosis, the left lungs were quickly immersed in 4% paraformaldehyde for 24 hours. Half of the lungs were dehydrated and embedded in paraffin followed by sectioning and staining with H&E, Masson’s trichrome, and picrosirius red. The remaining lungs were dehydrated with sucrose gradient sedimentation and embedded in OCT compound followed by cryosectioning for immunofluorescence staining. Paraffin sections of human healthy controls and IPF patients were offered by Qianfoshan Hospital, Shandong, PR China and were approved by the Institutional Review Board at Qianfoshan Hospital.

For immunohistochemistry analysis, tissue sections were treated with appropriate primary antibodies followed by incubation with HRP-conjugated secondary antibodies. The 3,3′-diaminobenzidine (DAB) substrate was used for direct visualization of the distribution of target proteins.

### Quantitative RT-PCR

Total RNA was extracted from either cell lysate or the snap-frozen right lungs of mice with TRIzol reagent. The cDNA was obtained using a HiScript Q RT SuperMix for qPCR kit (+gDNA wiper, Vazyme) according to the manufacturer’s instructions. One microgram of total RNA was incorporated in each reaction. The qRT-PCR gene expression analysis was performed using ChamQ Universal SYBR qPCR Master Mix reagent on Step One Plus (Applied Biosystems, USA). qRT-PCR data were normalized to GAPDH as a housekeeping standard. Fold changes of target mRNAs were analyzed using the 2^−ΔΔ*C*T^ method.

### Western blotting

Both MLFs and quick-frozen right lungs were processed in lysis buffer containing 1× protease inhibitor cocktail followed by centrifugation to remove the lysate. BCA protein assay was conducted to determine the total protein concentrations. Equal volume of the samples was loaded and separated by SDS–polyacrylamide gel electrophoresis, transferred to polyvinylidene difluoride membrane, and blocked with 5% BSA. The membranes were incubated overnight with primary antibodies followed by the incubation with appropriate secondary antibodies at room temperature. Electrochemiluminescence chromogenic substrate was added to visualize the target bands using a ChemiDoc imaging system (Bio-Rad, USA).

### Pulmonary function test

The experimental animals were anesthetized by intraperitoneal injection of pentobarbital. The anesthetized mice were intubated with a 14-gauge cannula. PFT was performed using a FlexiVent system (SCIREQ, Canada). Pulmonary function parameters were recorded for endpoint measurements.

### In vivo safety evaluation

To assess the in vivo toxicity, body weights were recorded every day and the survival of different treatment groups was analyzed. Blood was collected retro-orbitally at the end of the experiments for the ALT and AST measurements. The organ coefficient was calculated to assess the impact of different treatments on tissue alterations.

### Statistical analysis

All results are analyzed using GraphPad Prism software (version 8.2.1) and expressed as means ± SD. Statistical analysis was performed as indicated in the figure legends, and survival analysis was performed using the log-rank test. Nonparametric Spearman correlation coefficient was computed using GraphPad Prism software. Differences were considered statistically significant at *P* < 0.05. *P < 0.05, **P < 0.01, ***P < 0.001, and ****P < 0.0001.
