# Supplementary material for: Inhaled siRNA nanoparticles targeting IL11 inhibit lung fibrosis and improve pulmonary function post-bleomycin challenge
Source: Sci Adv. 2022 Jun 22;8(25):eabn7162. doi: 10.1126/sciadv.abn7162 (PMC9216512; doi:10.1126/sciadv.abn7162)
Supplement: Supplementary file 1 — Figs. S1 to S18 Table S1 [file sciadv.abn7162_sm.pdf]

Science Advances
AAAS

Supplementary Materials for

Inhaled siRNA nanoparticles targeting *IL11* inhibit lung fibrosis and improve pulmonary function post-bleomycin challenge

Xin Bai *et al.*

Corresponding author: Xiaoyang Xu, xiaoyang.xu@njit.edu; Xue-Qing Zhang, xueqingzhang@sjtu.edu.cn

Sci. Adv. **8**, eabn7162 (2022)
DOI: 10.1126/sciadv.abn7162

This PDF file includes:

Figs. S1 to S18
Table S1

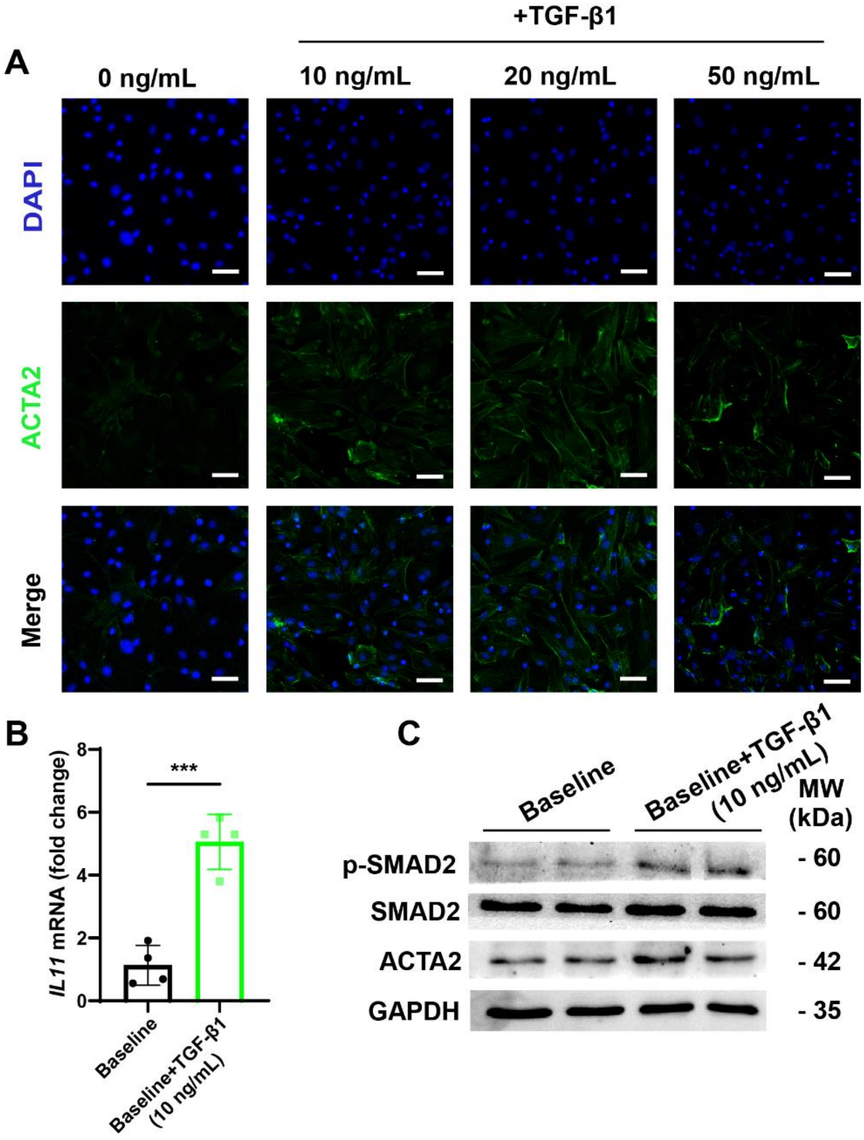

fig. S1. TGF-β1-induced fibroblast activation and *IL11* expression. (**A**) Representative immunofluorescence images of MLFs after treatment with 0, 10, 20 and 50 ng/mL TGF-β1. Blue and green fluorescence indicated nucleus and ACTA2, respectively (N=3). Scale bars, 50 μm. (**B**) Comparison of *IL11* gene expression level between baseline (PBS treatment) and TGF-β1 treatment (10 ng/mL) (N=4). (**C**) Western blotting of ACTA2, phosphorylation and total expression of SMAD2 in MLFs with or without TGF-β1 treatment (N=2). GAPDH was used as a housekeeping standard. Significant difference was assessed using a two-tailed unpaired Student's t test (**B**). Results are presented as mean ± SD. ***P<0.001.

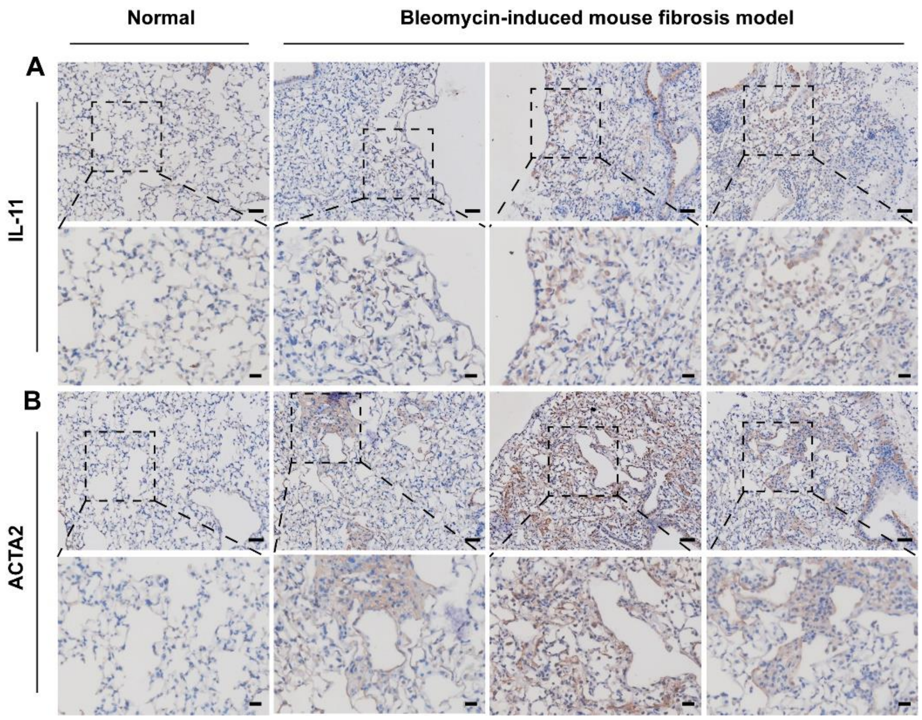

fig. S2. Immunohistochemistry staining of IL-11 and ACTA2 in lung tissues of bleomycin-induced fibrosis mouse model. (**A** and **B**) Representative images of IL-11 (**A**) and ACTA2 (**B**) in lung sections collected from healthy mouse (control, *N*=3) and the mouse model of bleomycin-induced pulmonary fibrosis (*N*=6) under different magnifications. Scale bars, up: 50 μm, bottom: 20 μm.

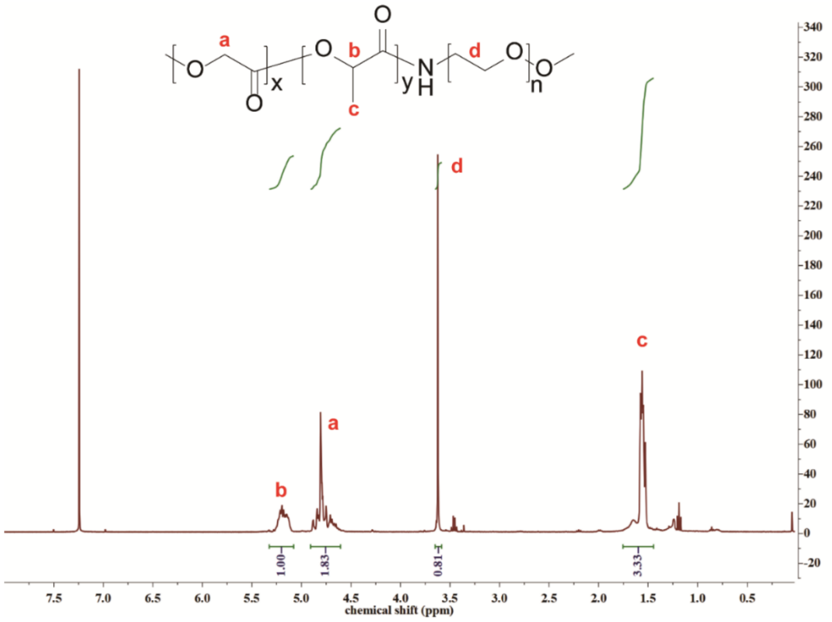

fig. S3. The ^{1}H NMR spectrum of PLGA-PEG.

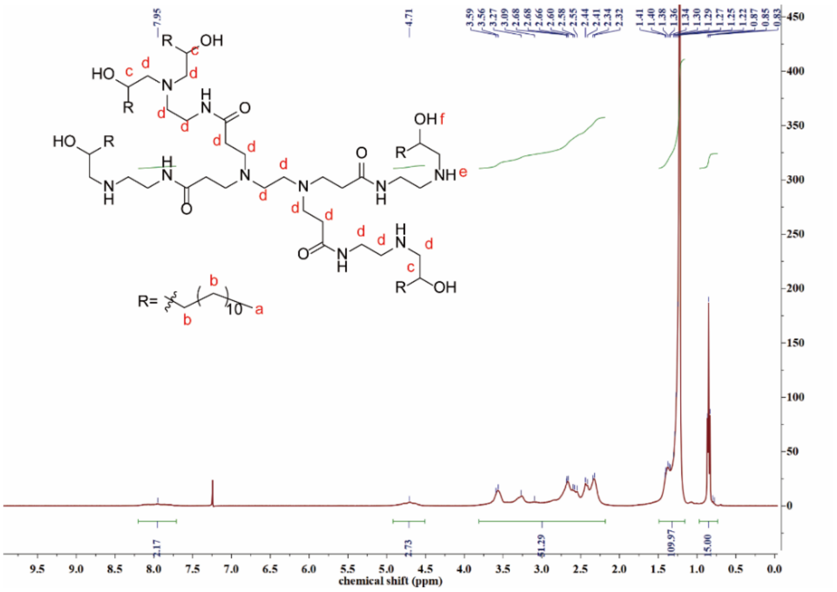

fig. S4. The ^{1}H NMR spectrum of G0-C14.

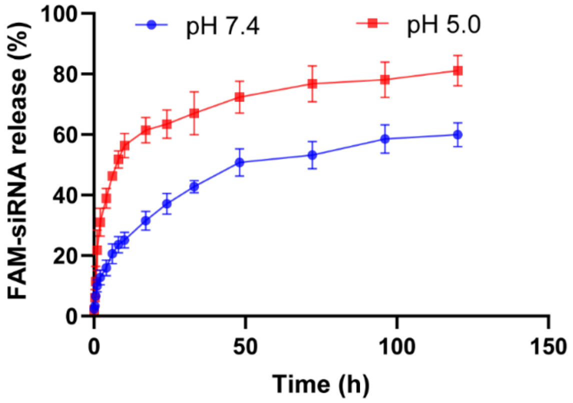

fig. S5. The release profile of siRNA@PPGC NPs in PBS of pH 7.4 and 5.0 (*N*=3).

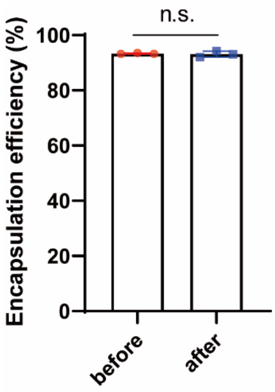

fig. S6. siRNA encapsulation efficiencies within NPs pre- and post- nebulization (*N*=3). Significant difference was assessed using a two-tailed unpaired Student's *t* test. Results are presented as mean ± SD. n.s., not significant, *P*>0.05.

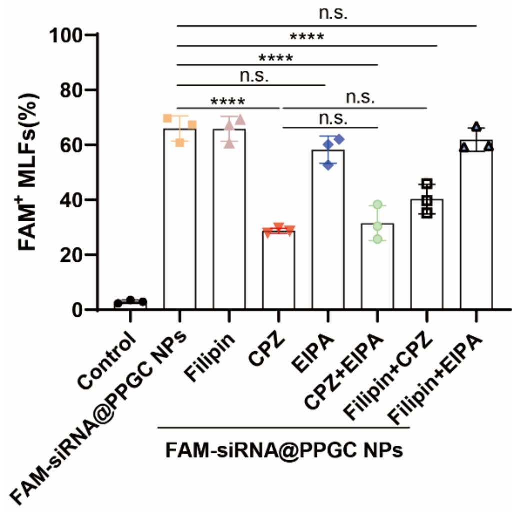

fig. S7. Cellular uptake mechanism of FAM-siRNA@PPGC NPs in MLFs (*N*=3). Significant difference was assessed using a one-way ANOVA with Tukey test. Results are presented as mean ± SD. ****P<0.0001, n.s., not significant, P>0.05.

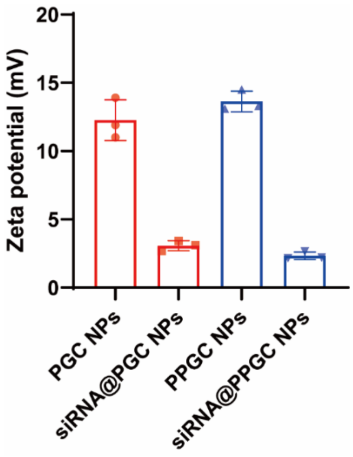

fig. S8. Surface zeta potential of PGC NPs, siRNA@PGC NPs, PPGC NPs, and siRNA@PPGC NPs (*N*=3).

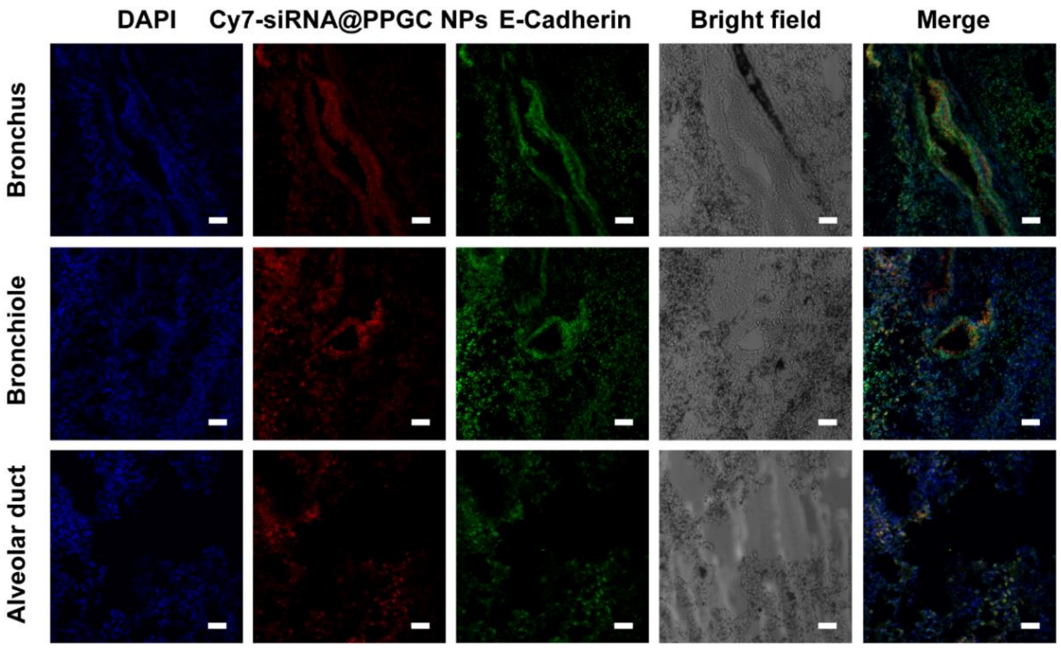

fig. S9. In vivo distribution of Cy7-siRNA@PPGC NPs in mouse lungs. Representative images show localization of Cy7-siRNA@PPGC NPs in the bronchus, bronchiole, and alveolar ducts (*N*=3). Blue, red, and green fluorescence indicated nuclei, NPs, and E-Cadherin, respectively. Scale bars, 50 μm.

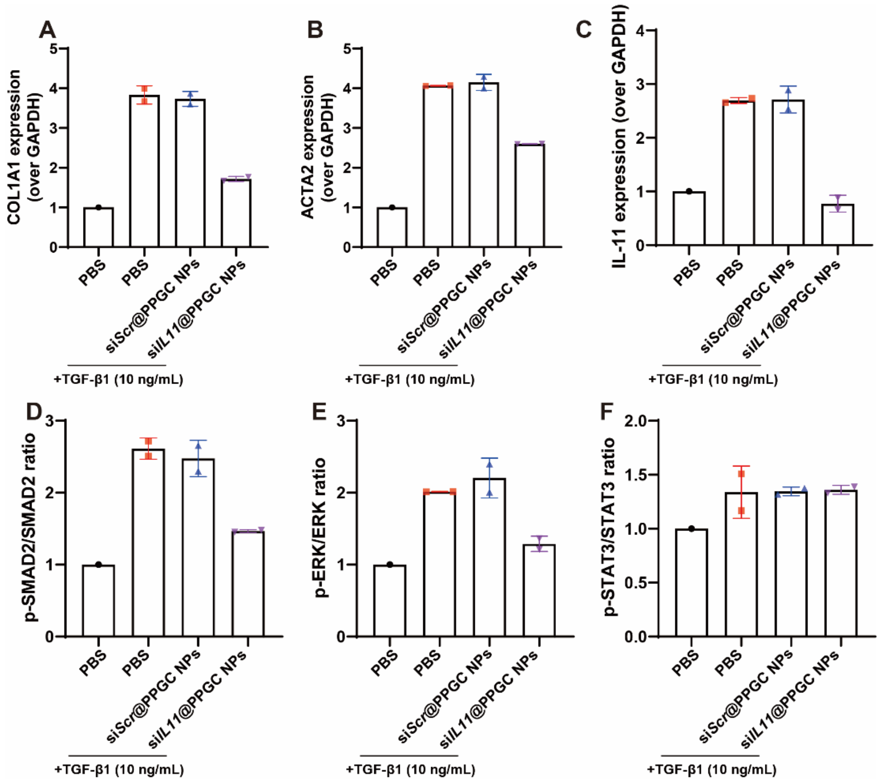

fig. S10. Densitometry analysis of western blots. The bands of COL1A1, ACTA2, IL-11, phosphorylation and total expression of SMAD2, ERK and STAT3 in Fig. 4D and 4E were analyzed with Image software (*N*=2).

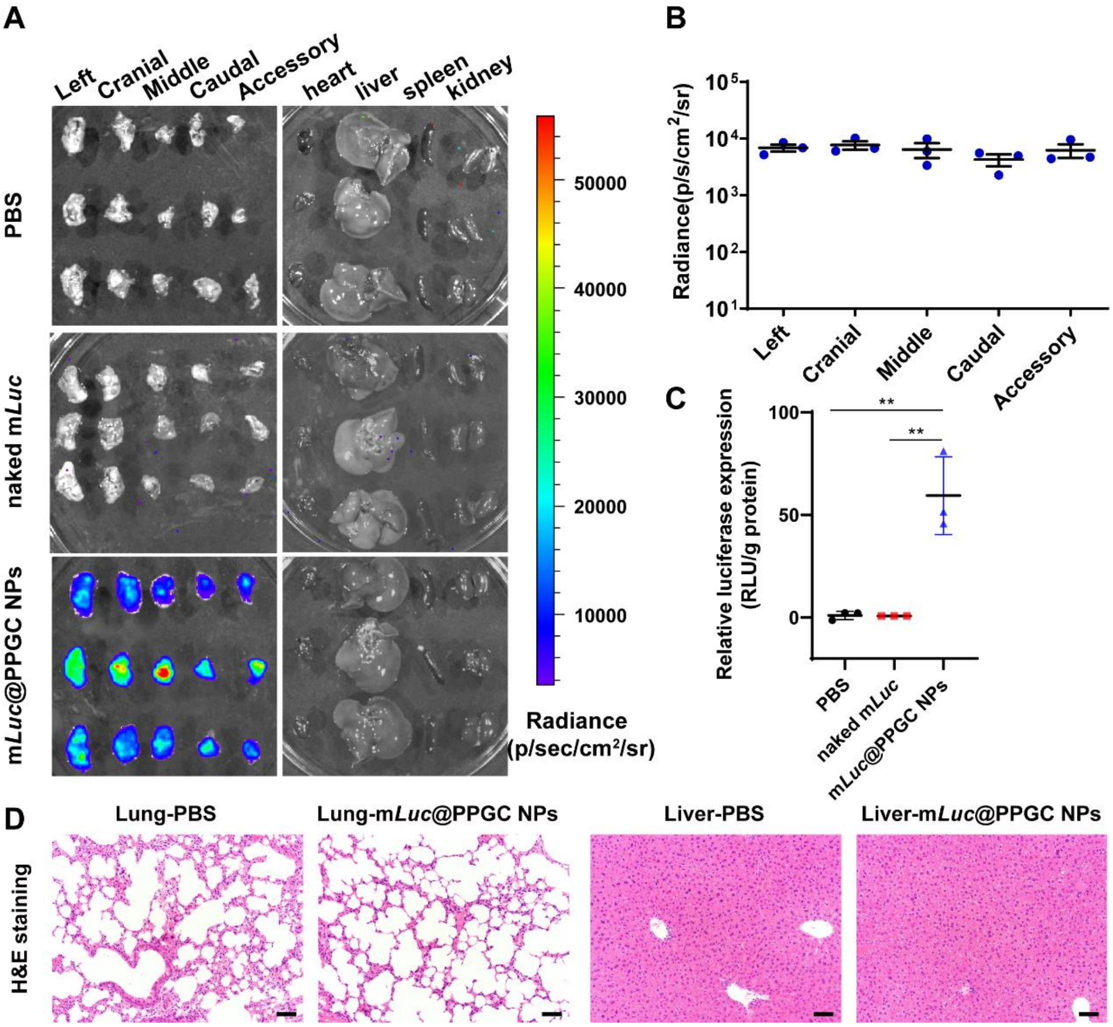

fig. S11. Transgene expression in mouse lungs after inhalation of nebulized m*Luc*@PPGC NPs. (**A**) Bioluminescence images of mouse lungs 24 h after inhalation of nebulized m*Luc*@PPGC NPs, naked m*Luc* solution, and PBS (N=3). (**B**) Uniform expression of luciferase in five lung lobes (N=3). (**C**) Relative luciferase expression in mouse lungs (N=3). (**D**) H&E staining of lung and liver tissues harvested 24 h post-nebulization. Scale bars, 50 μm (N=3). Significant difference was assessed using a one-way ANOVA with Tukey test. Results are presented as mean ± SD. **P<0.01.

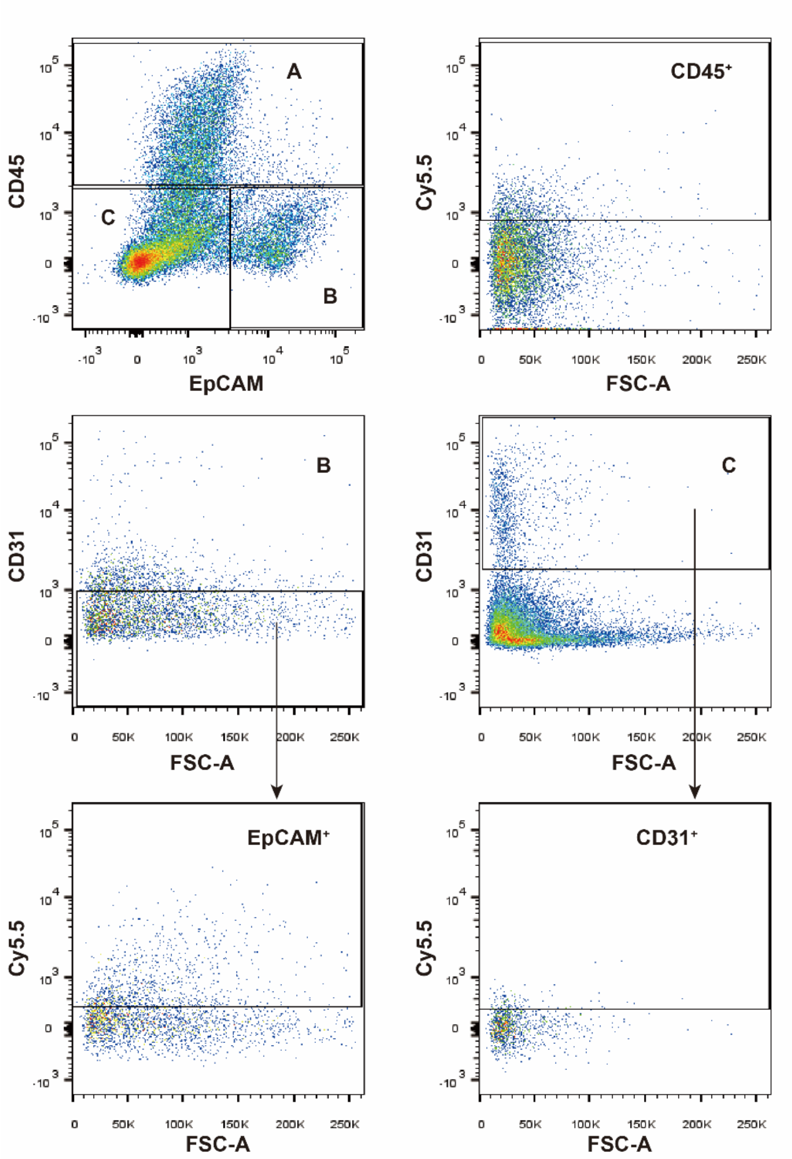

fig. S12. Gating strategy used for flow cytometry analysis of cell subtypes after aerosol delivery of Cy5.5-labeled si*IL11*@PPGC NPs. Markers for lung epithelial ($EpCAM^+$), endothelial ($CD31^+$) and immune ($CD45^+$) cells were used to distinguish cell subtypes.

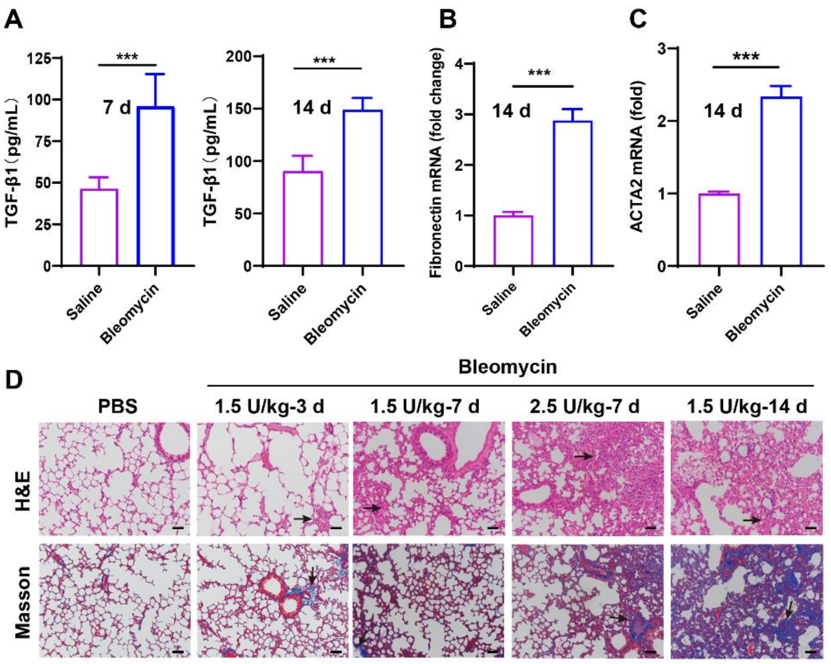

fig. S13. Establishment of bleomycin-induced fibrosis mouse models and dosage optimization. (**A**) Measurements of TGF-β1 level in BALF collected from healthy mice and fibrosis models on day 7 and day 14 after low dose of bleomycin (1.5 U/kg) challenge (*N*=5). (**B** and **C**) Gene expression levels of fibronectin (**B**) and ACTA2 (**C**) in bleomycin (1.5 U/kg)-induced fibrosis mouse models and healthy animals (*N*=3). (**D**) H&E and Masson staining of lung tissues from healthy mice and fibrosis models on day 3, day 7 or day 14 after bleomycin treatment at a dose of 1.5 U/kg or 2.5 U/kg (*N*=5). Scale bars, 50 μm. Significant difference was assessed using a two-tailed unpaired Student's *t* test. Results are presented as mean ± SD. ****P*<0.001.

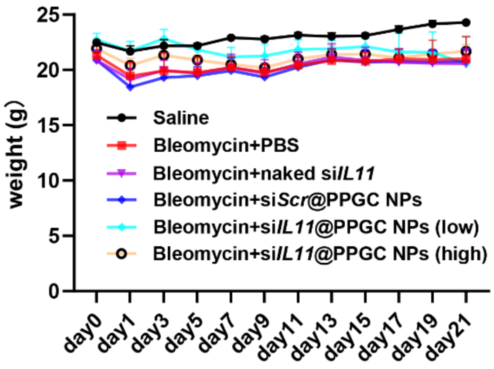

fig. S14. Weight changes of saline-treated healthy controls and bleomycin-induced fibrosis animals after treatment of PBS, si*Scr*@PPGC NPs, naked si*IL11*, or si*IL11*@PPGC NPs (*N*=5). Results are presented as mean ± SD.

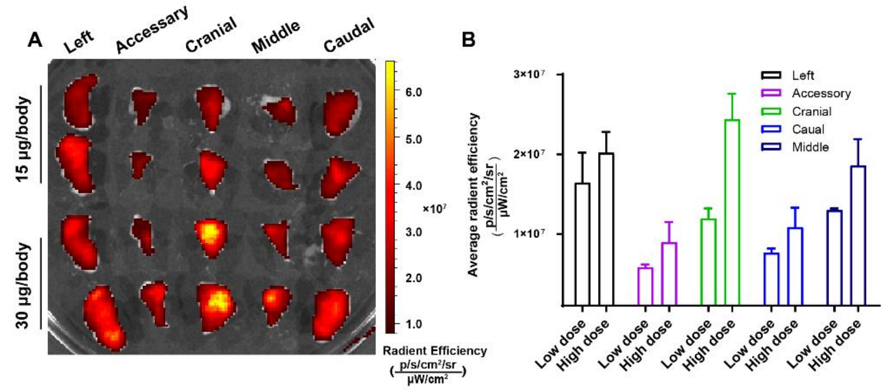

fig. S15. Dose-dependent accumulation of NPs in mouse lung tissues post inhalation of Cy7-si*IL11*@PPGC NPs. (**A**) Representative IVIS images of lungs at 24 h post-nebulization of Cy7-si*IL11*@PPGC NPs. **(B)** Quantitative analysis of fluorescence signal in left, accessory, cranial, middle, and caudal lobes (*N*=2). Results are presented as mean ± SD.

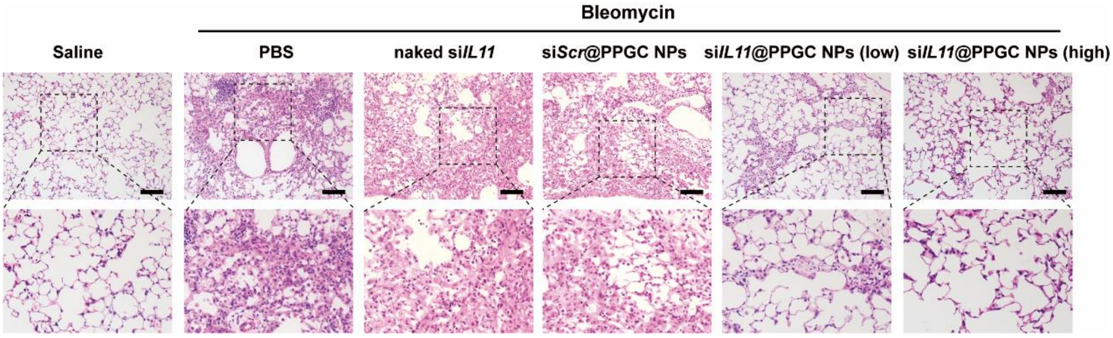

fig. S16. H&E staining of lung sections from groups treated with Saline, bleomycin+PBS, bleomycin+naked si*IL11*, bleomycin+si*Scr*@PPGC NPs, bleomycin+si*IL11*@PPGC NPs (low), bleomycin+si*IL11*@PPGC NPs (high). Scale bars, 50 µm.

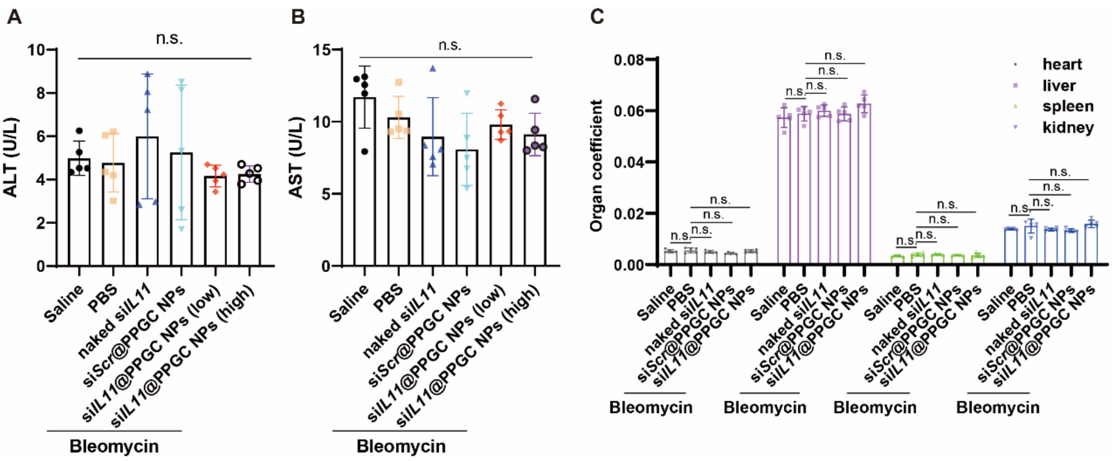

fig. S17. In vivo safety evaluation of saline-treated healthy controls and bleomycin-induced fibrosis animal models. (**A** and **B**) Analysis of serum ALT (**A**)

and AST (**B**) (*N*=5). (**C**) Organ coefficient of saline-treated healthy controls and bleomycin-induced fibrosis animal models after treatment with PBS, si*Scr*@PPGC NPs, naked si*IL11*, or si*IL11*@PPGC NPs (*N*=5). Significant difference was assessed using a one-way ANOVA with Tukey test. Results are presented as mean ± SD. n.s., not significant, *P*>0.05.

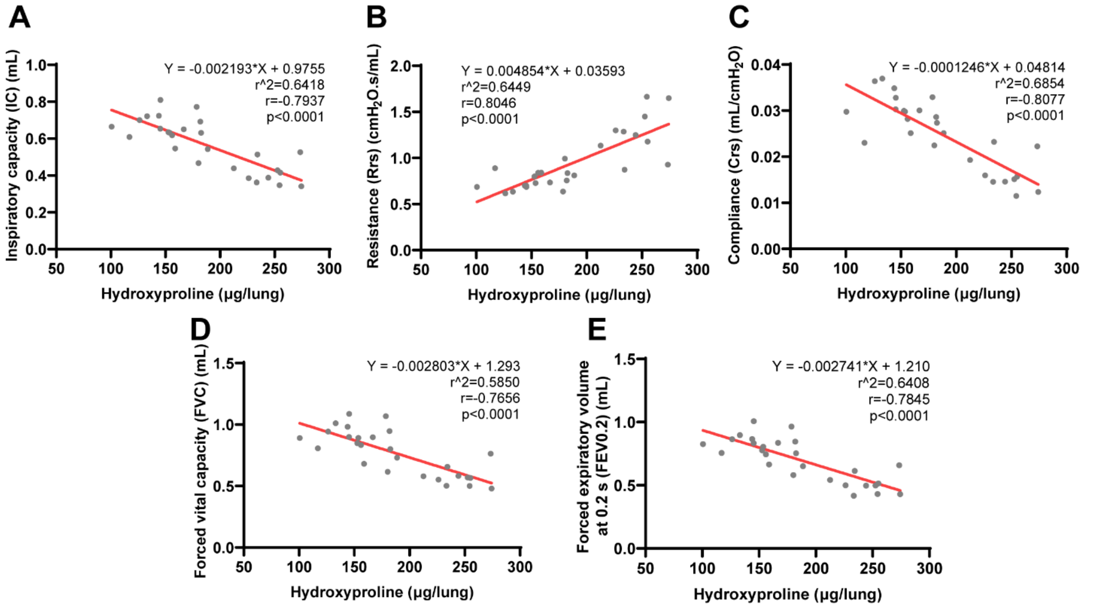

fig. S18. Correlation analysis between hydroxyproline content and pulmonary function parameters. Investigated parameters contain IC (**A**), Rrs (**B**), Crs (**C**), FVC (**D**) and FEV0.2 (**E**) (*N*=27). Correlation coefficient (r) was assessed based on a nonparametric Spearman correlation analysis.

Table 1. Sequences of si*IL11*s.

Number	Sense (5'-3')	Anti-sense (5'-3')
1	GCUGUUCUCCUAACCCGAUTT	AUCGGGUUAGGAGAACAGCTT
2	GCUGGGACAUUGGGAUCUUTT	AAGAUCCCAAUGUCCCAGCTT
3	GCCGUUUACAGCUCUUGAUTT	AUCAAGAGCUGUAAACGGCTT
